# RAFT step-growth polymerization *via* the Z-group approach and deconstruction by RAFT interchange†

**DOI:** 10.1039/d3sc06736j

**Published:** 2024-02-23

**Authors:** Jiajia Li, Joji Tanaka, Qing Li, Claire Jing Jing Wang, Sergei Sheiko, Samantha Marie Clouthier, Jian Zhu, Wei You

**Affiliations:** a State and Local Joint Engineering Laboratory for Novel Functional Polymeric Materials, Jiangsu Key Laboratory of Advanced Functional Polymer Design and Application, Department of Polymer Science and Engineering, College of Chemistry, Chemical Engineering and Materials Science, Soochow University Suzhou 215123 China; b Department of Chemistry, University of North Carolina at Chapel Hill NC 27599 USA joji@email.unc.edu wyou@unc.edu

## Abstract

Recycling vinyl polymers is essential to mitigate the environmental impact of plastic waste. However, typical polymerization strategies to construct vinyl polymers lack the ability to incorporate degradable linkers throughout the main chain. We report a RAFT step-growth polymerization through the Z-group approach that is directly carried out by using a common class of symmetric trithiocarbonate based RAFT agents and commercially available bismaleimide monomers. Such synthesized RAFT step-growth polymers contain embedded RAFT agents in every structural unit, allowing chain expansion of the step-growth backbone *via* controlled chain growth to yield linear multiblock (co)polymers. These polymers can undergo deconstruction *via* the RAFT interchange process with exogeneous RAFT agents, generating smaller uniform species with narrow molecular weight distribution. In addition, the telechelic bifunctional RAFT agent nature after deconstruction allows repolymerization, showing a promising method for recycling common vinyl polymers.

## Abstract 2

RAFT step-growth polymerization *via* the Z-group approach was developed, offering a facile method to prepare deconstructable (multiblock) polymers by combining RAFT chain-growth polymerization and RAFT interchange.

## Graphical abstract

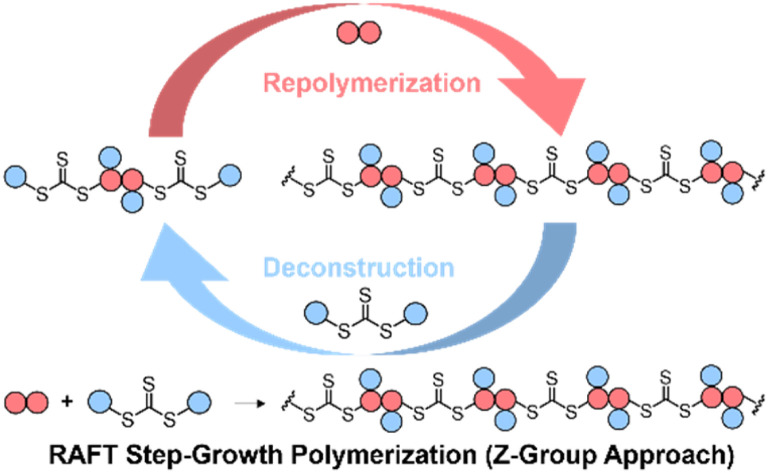

## Introduction

The C–C single bonds in vinyl polymers offer excellent stability for the intended applications, yet these polyethylene-like polymers are notoriously difficult to degrade, posing grand challenges in recycling these polymers and when degradation is desirable (*e.g.*, drug delivery).^1–3^ Various strategies have been developed to overcome this challenge, including copolymerization with degradable monomers,^4–18^ introduction of degradable groups by combining with step-growth polymerization,^19–24^ depolymerization to monomers under special conditions,^25–31^ and degradation to oligomers catalyzed by special reactions.^32–35^ Among them, introducing labile carbon-heteroatom bonds (*e.g.*, ester) into vinyl polymers *via* copolymerization of a second monomer containing such labile bonds is one of the most efficient methods.^2,3^ However, the insertion of these special monomers is usually random/statistical due to their different reactivities from the vinyl monomers; such synthesized “random” copolymers would thus result in uncontrolled degradation into a mixture of oligomeric species.^3^ For instance, Kiel *et al.* recently copolymerized styrene with a thionolactone (dibenzo[*c*,*e*]-oxepine-5(7*H*)-thione, DOT) to successfully impart degradation to polystyrene (PS);^7^ yet the degraded PS showed a molar mass dispersity around 1.9, due to the statistical nature of their copolymerization.

To ensure a homogeneous degradation, it would require uniform insertion of these degradable labile carbon-heteroatom bonds into the main chain. One such method has been elegantly demonstrated by Uchiyama *et al.* where they applied controlled cationic copolymerization of vinyl ethers with a 7-membered cyclic thioacetal to achieve a rather uniform degradation of poly(vinyl ether)s.^36^ Furthermore, these thioacetals can serve as in-chain dormant species to enable the controlled growth of internal segments of poly(vinyl ether)s with cationic polymerization of different vinyl ethers *via* the degenerative chain-transfer (DT) mechanism; such multiblock copolymers can degrade into homogeneous diblock copolymers. However, this method is limited by the monomer scope of cationic polymerization; by contrast, radical polymerization is generally applicable to a wide scope of vinyl monomers and is much more tolerant to various functional groups and solvents. Thus it would be desirable to design similar strategies to achieve homogeneous degradation, in particular, for biological applications.^3^

Indeed, installation of in-chain dormant species in polymers has been explored *via* reversible addition–fragmentation chain transfer (RAFT) polymerization, one of the most versatile reversible deactivation radical polymerization (RDRP).^37–39^ Such polymers can be prepared *via* either chain-growth copolymerization with cyclic trithiocarbonates^40–43^ or step-growth polymerization of a bifunctional reagent tethered by a trithiocarbonate core.^44–47^ Subsequent RAFT controlled chain-growth polymerization of vinyl monomers would produce internal segments between in-chain dormant species, and such synthesized vinyl polymers could then degrade into uniform oligomers (*i.e.*, “freed” internal segments). However, the syntheses of these cyclic chain transfer agents (CTAs) or RAFT agents with special functional groups (with some exceptions^46,47^) are rather complicated, limiting the scale-up and wide applications. In addition, there are limited strategies for repolymerization of degraded units following the degradation,^7^ yet such repolymerizations are required for closed loop recycling. We noticed that in-chain RAFT agents in polymer networks have been exploited to achieve self-healing materials *via* photoinduced covalent bond rearrangement through the RAFT process (or RAFT interchange);^48–53^ these intriguing results prompted us to explore the dynamic RAFT interchange for recycling polymers with in-chain CTAs; ideally, such polymers could be prepared in a simple and highly efficient manner.

**Fig. 1:**
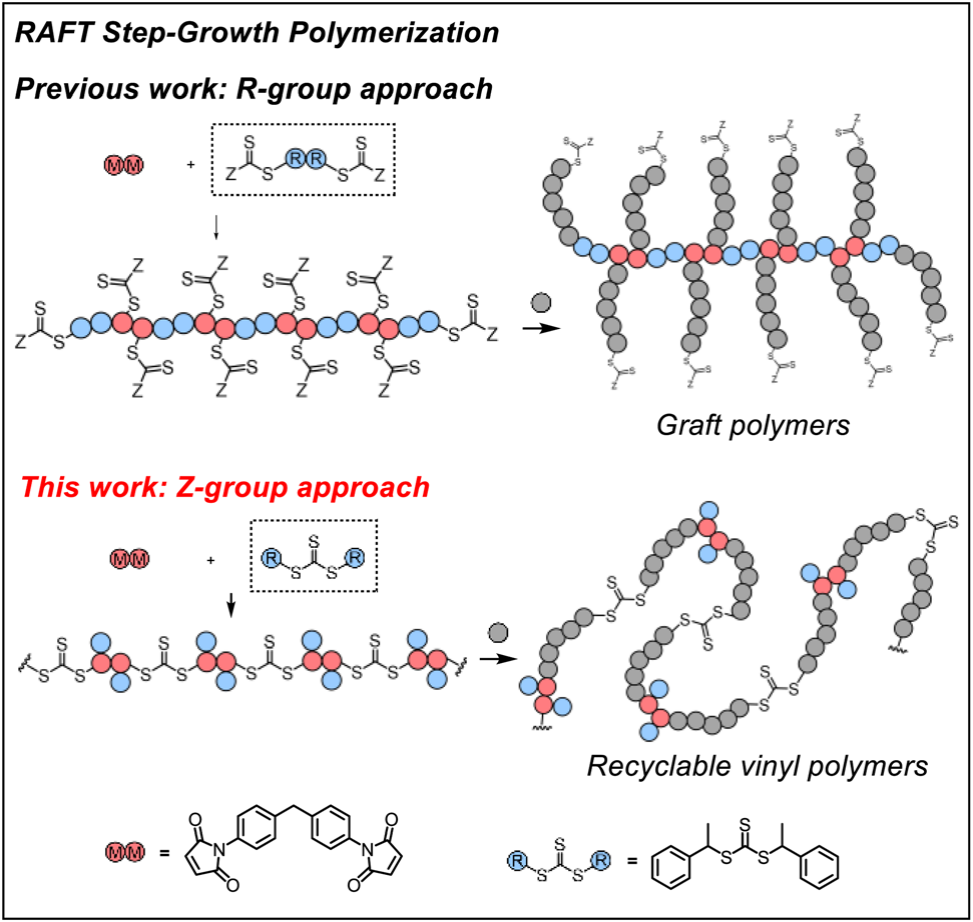
RAFT step-growth polymerization *via* the R-group approach or the Z-group approach.

Previously, we demonstrated RAFT step-growth polymerization (or polyaddition, suggested by IUPAC^54,55^) by exploiting bifunctional reagents bearing a monomer and CTA functionality that can generate high yields of single unit monomer inserted (SUMI)^56^ CTA adducts under stoichiometrically balanced conditions.^57–62^ In our earlier reports, the CTA was tethered with the monomer (*i.e.*, for AB step-growth) or with the same CTA (*i.e.*, for A_2_ + B_2_ step-growth) through the R-group, which yields a polymer backbone with thiocarbonylthio units appended as the side chains on each structural unit. This unique configuration allows subsequent graft polymerization from the step-growth backbone (Fig. 1). We envisioned that RAFT step-growth can also be achieved by tethering the CTA through the Z-group, or more simply by employing a symmetric trithiocarbonate that bears two R-groups as the B_2_ for A_2_ + B_2_ step-growth polymerization. It is important to clarify that, although the Z-group is not explicitly presented in Fig. 1, the implied Z-group is the –SR group opposite the polymerizing side of the RAFT agent. By taking advantage of this proposed RAFT step-growth *via* the Z-group approach, herein we report a simple approach with easy-to-prepare or commercially available monomers to construct a polymer with uniformly installed RAFT agents, which can be chemically recycled through RAFT interchange with exogenous RAFT agents. Additionally, in contrast to the former R-group approach, multi-segment vinyl polymers can be obtained *via* subsequent main chain expansion of the step-growth polymer with vinyl monomers through RAFT controlled chain-growth. Furthermore, deconstruction of such vinyl polymers through RAFT interchange with exogenous RAFT agents results in lower molecular weight vinyl polymer species with narrow molecular weight distribution; these species can be considered as a bifunctional macro-CTA for repolymerization through the RAFT step-growth *via* the Z-group approach, thereby closing the loop for chemically recycling vinyl polymers.

## Results and discussion

### Mechanistic difference between the ‘R-group approach’ and ‘Z-group approach’ for RAFT step-growth polymerization

**Fig. 2:**
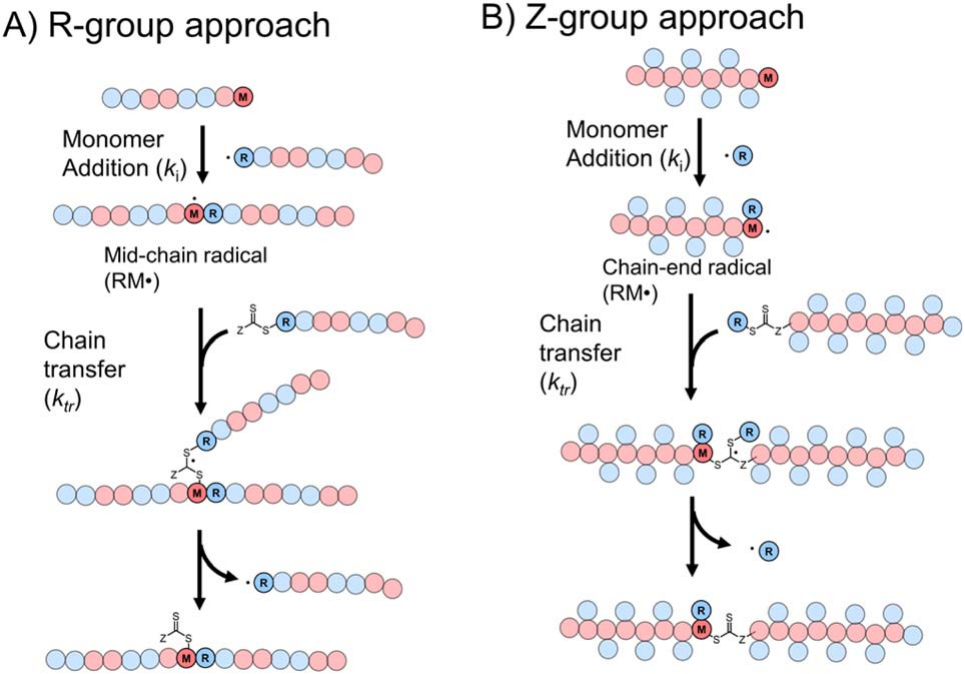
The difference between RAFT step-growth polymerization *via* (A) the R-group approach and (B) the Z-group approach.

Though RAFT step-growth polymerizations *via* the R-group approach and the Z-group approach proceed through an identical SUMI process, we highlight some key mechanistic differences in Fig. 2. For clarity, the thiocarbonyl thiol present for every repeat unit is not drawn in the illustration. In addition, specific to this work where a symmetric trithiocarbonate is used, the Z-group is a sulfur atom bearing a fragmentable group (polymer backbone or another R-group). With other classes of dithiocarbonyl thio based CTAs, the Z-group would be required to be tethered to another Z-group bearing a dithiocarbonyl thio unit *via* a non-fragmentable linker. The simplification of treating two groups of dithiocarbonyl thio units to a single core is unique to this class of symmetric trithiocarbonates.

While with both R-group and Z-group approaches, monomer addition (*k*_i_) and chain transfer (*k*_tr_) are key steps to complete the RAFT step-growth cycle, the difference between how two CTAs are tethered (R *vs.* Z) has implications in the radical intermediate species and the way the polymeric chains are formed. In the R-group approach, the R˙ species exists as a (macro)radical end-group that adds to the monomer end-group, forming the R–M˙ adduct as a mid-chain radical species (Fig. 2A). However, in the Z-group approach, the “free” R˙ species adds to a monomer end group, forming an R–M˙ adduct as a chain-end radical species (Fig. 2B). The R–M˙ adduct will then be added to a chain end CTA to form a 3-arm star like polymer as a chain transfer intermediate adduct in the R-group approach (Fig. 2A), while in the Z-group approach this intermediate resembles a linear chain (Fig. 2B). Finally, following fragmentation from the chain transfer intermediate adduct, the respective R˙ species is regenerated, concurrently forming the CTA backbone. We emphasize that a ‘stable’ main chain is formed upon an irreversible monomer addition step in the R-group approach (Fig. 2A), whereas a ‘dynamic’ mainchain is formed upon the reversible chain transfer step in the Z-group approach (Fig. 2B).

### RAFT SUMI model reaction

**Fig. 3:**
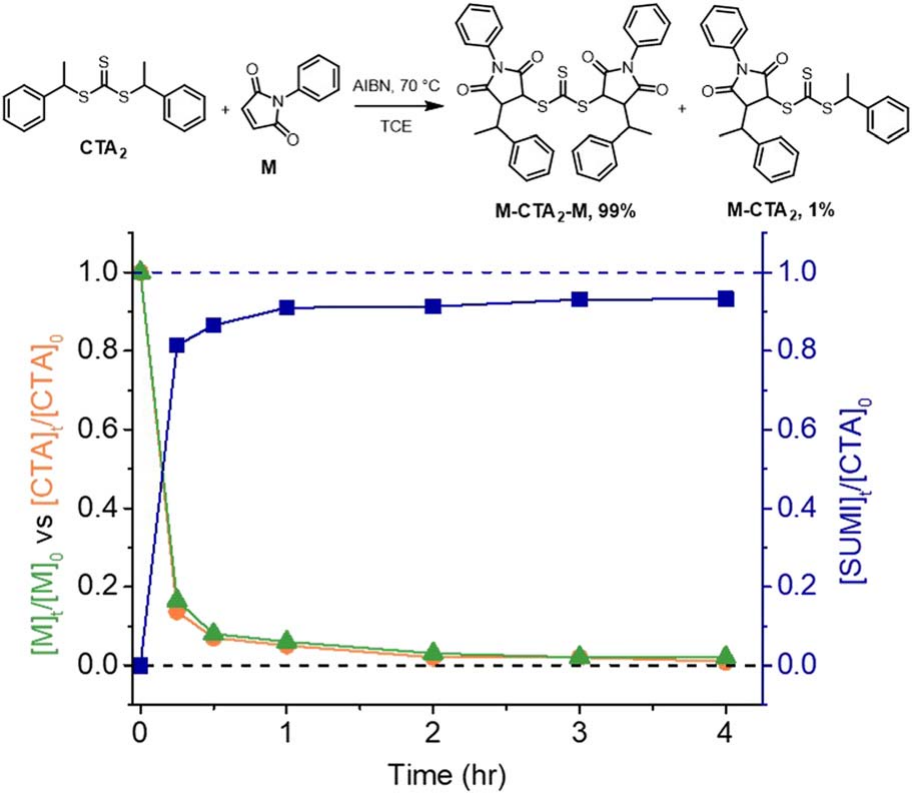
Kinetic analysis of the RAFT-SUMI model reaction between the RAFT agent and maleimide with [CTA_2_]_0_ : [M]_0_ : [AIBN]_0_ = 0.5 : 1:0.05 M in TCE at 70 °C. The RAFT-SUMI-CTA_2_ adduct (M-CTA_2_-M) yields (blue line) include both the dual SUMI-CTA_2_ adduct and mono-SUMI adduct (M-CTA_2_); the relative fraction of each species after 4 hours is included in the reaction scheme.

We chose a trithiocarbonate based CTA, bis(1-phenylethyl)trithiocarbonate (CTA_2_ in Fig. 3), to experimentally validate the RAFT step-growth polymerization *via* the Z-group approach, since this R-group has been previously reported to undergo an efficient RAFT-SUMI process with maleimides.^60,62^ In addition, we found this particular bifunctional CTA can be synthesized with high yield under mild conditions.^63^*N*-Ethyl maleimide was chosen as the monomer to attempt the model RAFT-SUMI reaction, to confirm if symmetrical trithiocarbonate based CTAs can efficiently yield SUMI-CTA adducts.^62^ In theory, one equivalent of CTA_2_ would consume two equivalents of monofunctional maleimide (Fig. 3). As shown in Fig. 3, S1 and Table S1,† near quantitative yield of the SUMI-CTA adduct was observed within 4 hours using a thermal initiator, 2,2-azobisisobutyronitrile (AIBN) at 70 °C, indicating the feasibility of this monomer/CTA pair for the RAFT step-growth polymerization.

### RAFT step-growth polymerization *via* the Z-group approach

**Fig. 4:**
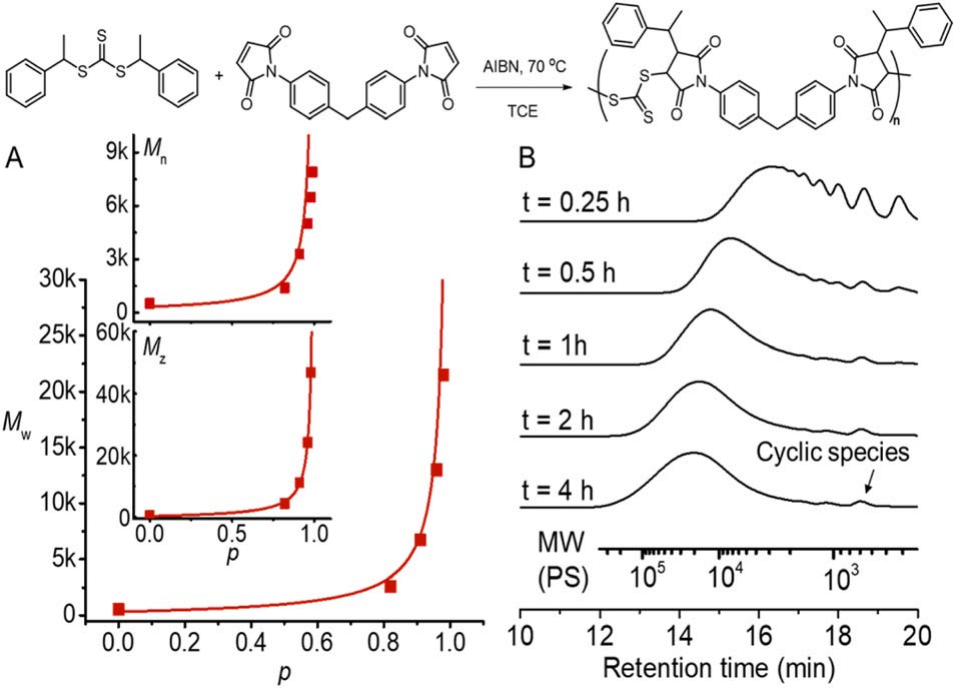
RAFT step-growth polymerization results using CTA2 and M_2_ with [CTA_2_]_0_ : [M_2_]_0_ : [AIBN]_0_ = 0.5 : 0.5 : 0.05 M in TCE at 70 °C. (A) Evolution of experimental *M*_n_, *M*_w_, and *M*_*z*_ determined by SEC analysis using polystyrene as a standard and extent of reaction (*p*) calculated from ^1^H NMR, plotted with theoretical molecular weight averages predicted without considering cyclization (see the ESI† for more information); (B) SEC traces of the obtained polymers at different time points.

We chose a low-cost and commercially available bismaleimide, 1,1′-(methylenedi-4,1-phenylene)bismaleimide, as the bifunctional monomer (M_2_) to demonstrate A_2_ + B_2_ type RAFT step-growth polymerization *via* the Z-group approach. Tetrachloroethane (TCE) was used as a solvent for its good solubility with maleimide monomers.^60^ Pleasingly, the evolution of number-average (*M*_n_), weight-average (*M*_w_), and *Z*-average (*M*_*z*_) molecular weight with the extent of reaction (*p*) (determined from ^1^H NMR, Fig. S2†) tracked very well with the theoretical values (Fig. 4A), validating the polymerization to proceed through linear step-growth molecular weight evolution. The lower experimental *M*_n_ values can be attributed to the formation of cyclic species observed in the SEC traces (Fig. 4B). The deviation of experimental *M*_w_ from theoretical values for balanced stoichiometry ([CTA_2_]_0_ : [M_2_]_0_ = 1 : 1) can be accounted for by the stoichiometric imbalance caused by external initiation (Table S2†).^62,64^ It is worth noting that irreversible radical termination events would also contribute to imbalanced stoichiometry.

### Recycling the RAFT step-growth polymers

**Fig. 5:**
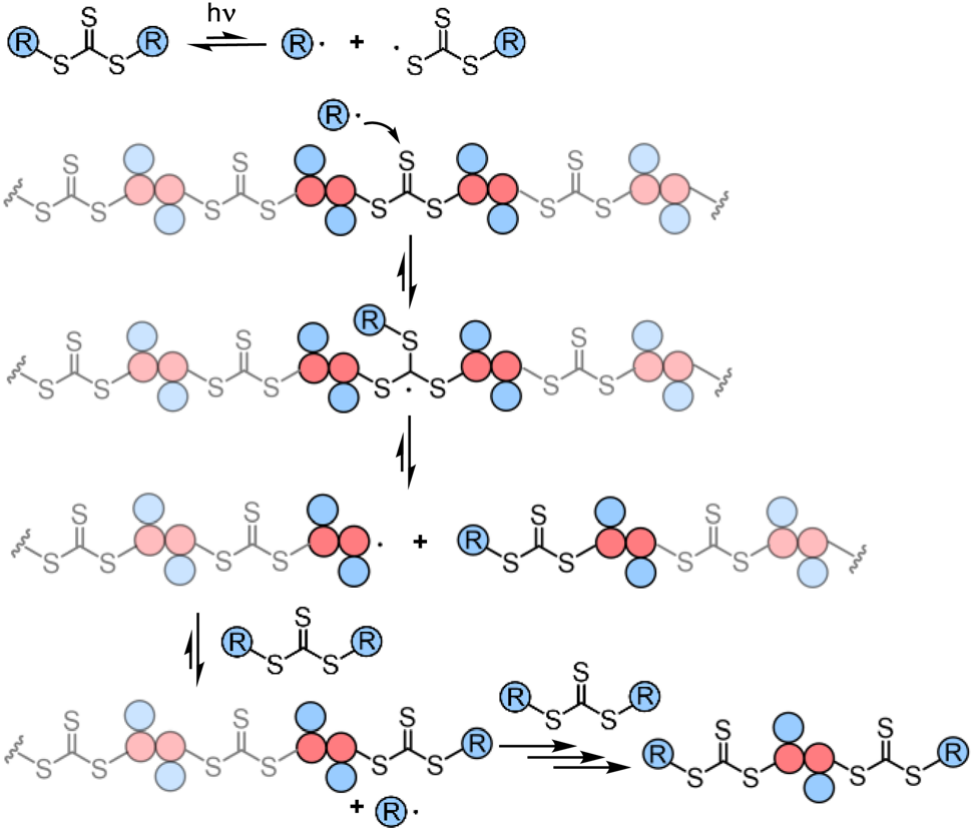
Proposed mechanism for the deconstruction of the backbone.

The presence of the in-chain trithiocarbonate (TTC) in the alternating copolymers prepared from RAFT step-growth polymerization *via* the Z-group approach would allow for both deconstruction through RAFT interchange and chain expansion through RAFT chain-growth polymerization. Before attempting the chain expansion from this polymer, we first examined its recyclability through RAFT interchange in the presence of exogenous RAFT agents. Mechanistically, as shown in Fig. 5, the exogenous CTA_2_ can undergo direct photolysis to generate a reactive carbon-centered radical, R˙, which can react with the in-chain TTC group through RAFT interchange, leading to fragmentation of the alternating copolymers. It is worth noting that this process is *reversible* and controlled by equilibrium; thus increasing the amount of the exogenous CTA_2_ will result in more complete deconstruction. On the other hand, owing to the dynamic nature of the in-chain TTC, reshuffling reactions of the polymer chain could also happen during the polymerization. Moreover, the direct photolysis of the carbon–sulfur bond in the backbone would promote the RAFT interchange process, which is independent of the structure difference between the backbone and exogenous CTA_2_ (Fig. S3†). The deconstruction process could also be considered as RAFT step-growth polymerization under imbalanced stoichiometry, which should in theory lead to a low degree of polymerization (DP).

**Fig. 6:**
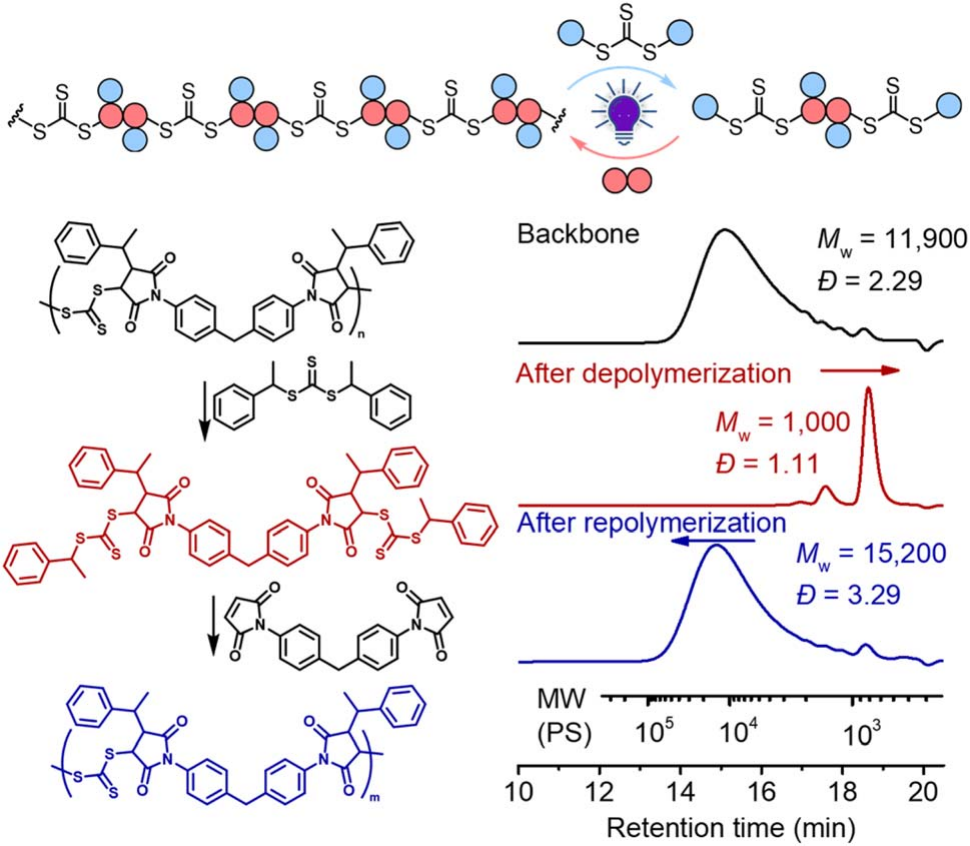
Deconstruction of the backbone with [TTC]_0_ : [CTA_2_]_0_ = 1 : 5 for 2 hours under irradiation of a 405 nm LED and (re)polymerization of the formed mixture with [CTA_2_]_0_ : [M_2_]_0_ = 1 : 1 for 96 hours under irradiation of a 405 nm LED.

As shown in Fig. 6, deconstruction of a backbone polymer (*M*_w_ = 11 900, *Đ* = 2.29) was conducted with CTA_2_ ([TTC]_0_ : [CTA_2_]_0_ = 1 : 5) under irradiation of a 405 nm LED. After 2 hours, the *M*_w_ decreased from 11 900 to 1000, consistent with the theoretical value of 994.2 for 3 structural units (2 × CTA_2_ + 1 × M_2_, Fig. 5), indicating successful deconstruction of this alternating copolymer. Moreover, it can be observed from ^1^H NMR that the signal of the benzylic *CH* (peak b, Fig. S4†) of the benzyl R group at the RAFT terminal increased and the signal of the *CH* (peak c, Fig. S4†) of the maleimide adjacent to trithiocarbonate progressed to a more defined splitting pattern after photoirradiation of the backbone with CTA_2_, further supporting the successful deconstruction. The minor peak in the higher molecular weight side in the SEC curve (Fig. 6) after deconstruction indicated incomplete deconstruction of the backbone. Decreasing the amount of CTA_2_ (*e.g.* [TTC]_0_ : [CTA_2_]_0_ = 1 : 1 or 1 : 2) resulted in more incomplete deconstruction, where more oligomers were observed (Fig. S5 and S6†). After the deconstruction, since the mixture consists of extra CTA_2_ and a newly generated bifunctional RAFT agent, adding M_2_ into this mixture would allow RAFT step-growth (re)polymerization. The exact amount of M_2_ can be calculated based on the amount of previously added CTA_2_ during the deconstruction. Indeed, successful repolymerization was achieved through both a photoiniferter method (Fig. 6 and S7–S9†) and thermally-induced method (Fig. S10†).^59^

### Deconstruction of (multiblock) vinyl polymers

**Fig. 7:**
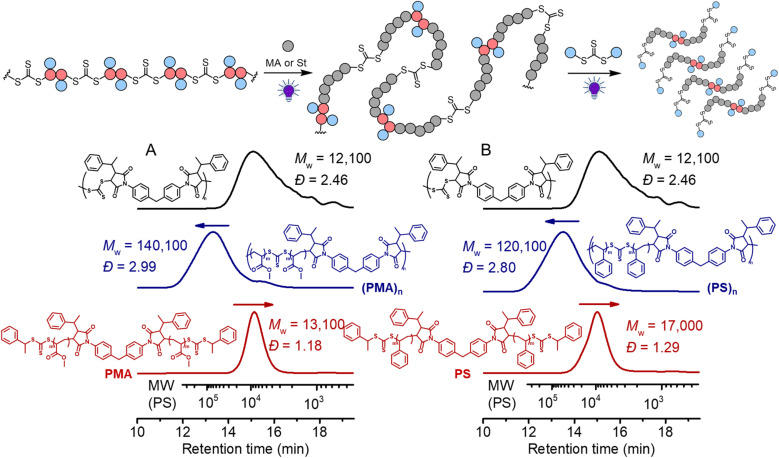
Black line: the backbone (*M*_w_ = 11 800); blue line: chain expansion under 405 nm light with [monomer]_0_ : [TTC]_0_ = 500 : 1; red line: after deconstruction of the obtained polymers with [TTC]_0_:[CTA_2_]_0_ = 1 : 10 for 24 hours; (A) using MA as a monomer at conv.% = 18.3%; (B) using St as a monomer at conv.% = 26.4%.

We next subjected this alternating copolymer (*i.e.*, the backbone) to chain expansion experiments with methyl acrylate (MA) and styrene (St), respectively, *via* photoiniferter RAFT polymerization under UV-irradiation of 405 nm.^65^ To avoid copolymerization of the possible terminal maleimide from the precursor step-growth polymer during the chain expansion, we applied a step-growth polymerization with an imbalanced stoichiometry at [CTA_2_]_0_ : [M_2_]_0_ = 1 : 0.9, which in theory should consume all bismaleimides during the step-growth. ^1^H NMR analysis of the obtained polymer (*M*_w_ = 12 100, *Đ* = 2.46) (Fig. S11†) disclosed that the signal assigned to the double bond in maleimide (peak d) at around 6.8 ppm nearly completely disappeared and the benzylic C*H* proton (peak i) was clearly visible at the chain end, supporting the anticipated alternating copolymer terminated by the CTA end group (structure in Fig. S11†). The targeted DP of the vinyl polymer was controlled by the molar ratio between the monomer and the in-chain TTC and the reaction time. As depicted in Fig. 7A, the SEC trace showed a clear shift after chain expansion with MA (the blue line, monomer conversion = 18.3% from ^1^H NMR), with *M*_w_ increasing from 12 100 to 140 100. The low molecular weight peak (though minor) is likely caused by the chain expansion of the cyclic species. Triple detection SEC (dRI, LS, VS) analysis was further employed for characterization of the polymers before and after chain expansion (Fig. S12 and S13†). The Mark–Houwink plots showed an *α* value of 0.66 for the backbone polymer and 0.677 for the PMA, consistent with the hydrodynamic volume as a function of molecular weight distribution for linear polymers.^66^ This contrasts to the case of subsequent RAFT chain-growth polymerization from RAFT step-growth polymers prepared by the R-group approach, where the *α* value decreases as the obtained graft copolymers behave more compact in solution.^62^ Furthermore, following the deconstruction *via* RAFT interchange with CTA_2_, a more symmetrical SEC trace was observed with narrow molecular weight distribution (the red line in Fig. 7A). Pleasingly, the experimentally determined *M*_n_ of 11 100 (from conventional SEC analysis with polystyrene calibration in THF, see the ESI† for SEC information) for the PMA segments after deconstruction was consistent with the theoretical value of 8900 (*M*_n,th_ = ([MA]_0_/[CTA]_0_) × *M*_MA_ × conv.% + *M*_**M**_2__ + 2 × *M*_**CTA**_2__). These results demonstrated the successful chain expansion of the backbone and its subsequent deconstruction into well-defined PMA segments. The chain expansion of the backbone was also carried out with St to prepare degradable polystyrene (PS). As depicted in Fig. 7B, successful chain expansion was observed with molecular weight increasing from 12 100 to 120 100 (blue line, monomer conversion = 26.4%). Meanwhile, the Mark–Houwink plots showed an *α* value of 0.655 (Fig. S14†), indicative of a linear PS as expected. The molecular weight of PS segments (*M*_n_ = 13 200, *Đ* = 1.29) after deconstruction was also consistent with the theoretical value of 14 700 (*M*_n,th_ = ([St]_0_/[CTA]_0_) × *M*_St_ × conv.% + *M*_**M**_2__ + 2 × *M*_**CTA**_2__). We have also performed the controlled experiment of PS in the absence of CTA_2_, and the reshuffling of backbone trithiocarbonate resulted in minor cyclization (Fig. S15†).

**Fig. 8:**
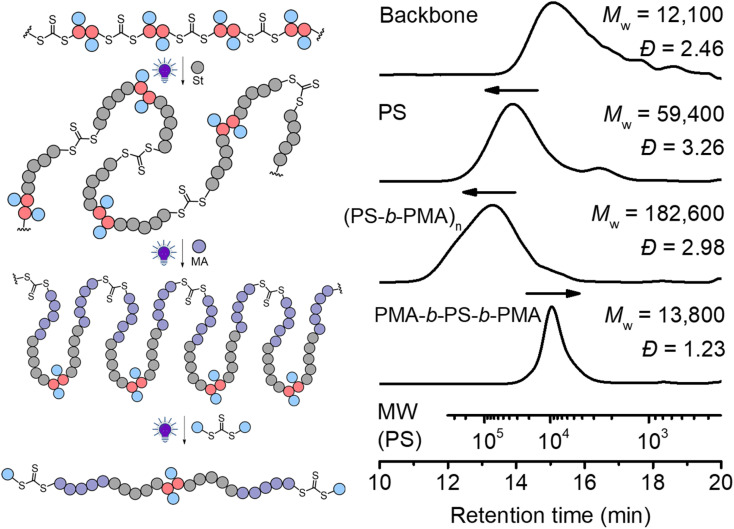
Chain expansion with St was carried out under 405 nm light with [St]_0_ : [CTA]_0_ = 330 : 1, conv.% = 12.7%; chain expansion with MA was carried out under 405 nm light with [MA]_0_ : [CTA]_0_ = 330 : 1, conv.% = 23.2%; deconstruction of the obtained polymer was carried out with [TTC]_0_ : [CTA_2_]_0_ = 1 : 10 under 405 nm light for 24 hours.

In traditional RAFT chain-growth polymerization, multiple chain extensions are required to prepare multiblock copolymers (typically generating one block per chain extension).^67^ By exploiting the in-chain RAFT agent, multiblock copolymers can be easily prepared by simple chain expansions from the step-growth backbone. As shown in Fig. 8, we demonstrate this by first chain expansion of a backbone (*M*_w_ = 12 100) with St (*M*_w_ = 59 400), followed by the second chain expansion with MA (*M*_w_ = 182 600), which results in multiblock copolymer of P(PS-*b*-PMA)_*n*_. The Mark–Houwink plots of the PS and multiblock showed *α* values of 0.69 and 0.617, respectively (Fig. S16†), confirming the formation of linear polymers. After deconstruction of the multiblock copolymer, a decreased *M*_w_ of 13 800 (from 182 600 of the multiblock copolymer) was observed with a narrow molecular weight distribution (*Đ* = 1.23), indicating a successful deconstruction of the multiblock copolymer to segments of controlled lengths (consisting of a triblock copolymer PMA-*b*-PS-*b*-PMA). Furthermore, the experimental number average molecular weight (*M*_n_ = 11 200, *Ð* = 1.23) matched well with the theoretical value of 12 900 predicted by the monomer conversion (*M*_n,th_ = ([St]_0_/[CTA]_0_) × *M*_St_ × conv.% (St) + ([MA]_0_/[CTA]_0_) × *M*_MA_ × conv.% (MA) + *M*_**M**_2__ + 2 × *M*_**CTA**_2__).

### Recycling vinyl polymers

**Fig. 9:**
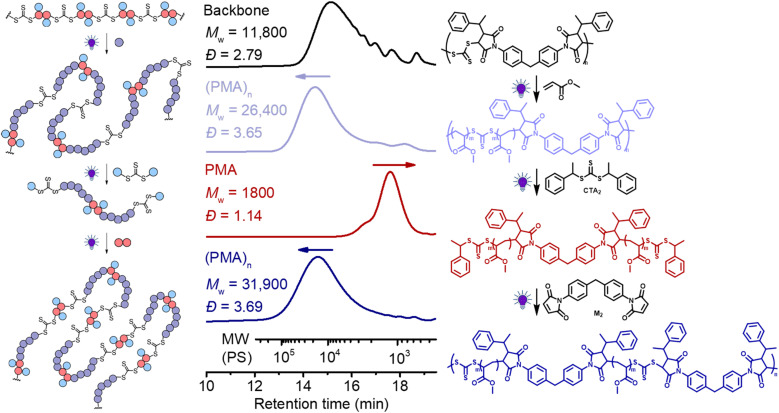
Recycling PMA through RAFT step-growth polymerization and deconstruction. Black line: the backbone; purple line: chain expansion with MA by RAFT chain-growth under 405 nm light with [MA]_0_ : [TTC]_0_ = 50 : 1, conv.% = 18.0%; red line: deconstruction of the obtained polymer under 405 nm for 24 h with [TTC]_0_ : [CTA_2_]_0_ = 1 : 10; blue line: repolymerization of PMA under 405 nm for 96 h with [PMA]_0_ : [M_2_]_0_ = 1 : 1.

Finally, we explored the iterative cycles of repolymerization and deconstruction to recycle vinyl polymers. We first examined (PMA)_*n*_ using the same bifunctional CTA (CTA_2_) for the deconstruction and bis-maleimide (M_2_) for repolymerization. We chose a polymer (after chain expansion with MA) having relatively low molecular weight PMA segments (Fig. 9, purple line, *M*_w_ = 26 400, *Đ* = 3.65), to allow repolymerization of the degradants (after deconstruction) at sufficiently high molar concentrations, because a high concentration is required to achieve high molecular weight.^62^ It is worth noting that ^1^H NMR reveals shifts in the C*H* protons next to the trithiocarbonate following chain expansion of the backbone with MA: the first two sets of signals (end-group – benzyl C*H*, peak a at 5.25 ppm *vs.* in-chain maleimidic C*H*, peak b at 4.70 ppm, Fig. S17†) of the step-growth backbone converted to single species of C*H* in-chain termini of PMA segments at 4.82 ppm (peak c, Fig. S17†) after chain expansion of MA. These observable differences in ^1^H NMR became convenient in analyzing each step of the recycling process (*vide infra*).

As depicted in Fig. 9, (PMA)_*n*_ with relatively low molecular weight (purple line, *M*_w_ = 26 400, *Đ* = 3.65) was prepared by chain expansion of the backbone (black, *M*_w_ = 11 800, *Đ* = 2.79); after deconstruction with CTA_2_, this (PMA)_*n*_ degraded into PMA with low molecular weight and narrow dispersity (red line, *M*_w_ = 1,800, *Đ* = 1.14). After deconstruction, it should be pointed out that each terminal RAFT agent of the PMA has two different fragmentations, including a benzyl R-group and a secondary ester macro-R-group, which can be observed by ^1^H NMR (approximately 50 : 50 distribution of *H*_d_ and *H*_e_, Fig. S17†). For (re)polymerization of the PMA *via* RAFT step-growth, a selective reaction between only one of the two fragmentations at each chain end is required to avoid the crosslinking reaction. In theory, selective (re)polymerization through the macro-R-group would lead to the RAFT step-growth *via* the R-group approach, whilst to achieve step-growth *via* the Z-group approach, selective repolymerization must occur through the other R-group. In our case, we chose the bismaleimide (M_2_) for the repolymerization because of its preferable RAFT-SUMI reaction with the benzyl R-group than the secondary ester R-group.^62^ As shown in Fig. 9 (blue line), the *M*_w_ increased from 1800 to 31 900 after (re)polymerization with M_2_, demonstrating the success of this approach. Furthermore, ^1^H NMR analysis revealed the expected disappearance of the C*H* peak of the benzyl R-group next to the trithiocarbonate after repolymerization (Fig. S17†). Moreover, this polymer can be deconstructed again by adding CTA_2_ through the RAFT interchange (red line, Fig. S18†); interestingly, a peak at the low molecular weight side after deconstruction was observed, which was assigned to the adduct of M_2_ and CTA_2_ generated during the RAFT interchange (Fig. S19†). Unfortunately, separating this adduct from the degraded low molecular weight PMA was found to be challenging as it was more insoluble than PMA during purification by precipitation. Nonetheless, successful (re)polymerization can still be achieved by adding M_2_ to this mixture (blue line, Fig. S18†), though likely leading to a random copolymer containing segments from the original backbone and from the (PMA)_*n*_.

We also applied the same strategy for recycling PS. However, we noticed after deconstruction with CTA_2_ that each terminal RAFT agent of the PS has two phenyl fragmentations (Fig. 7B), which would lead to crosslinking during the (re)polymerization, due to the lack of selectivity. Therefore, a RAFT agent with a tertiary ester R group (CTA_2E_) was synthesized and used (instead of CTA_2_) for the deconstruction, aiming to subsequently achieve selective repolymerization. As shown in

**Fig. 10:**
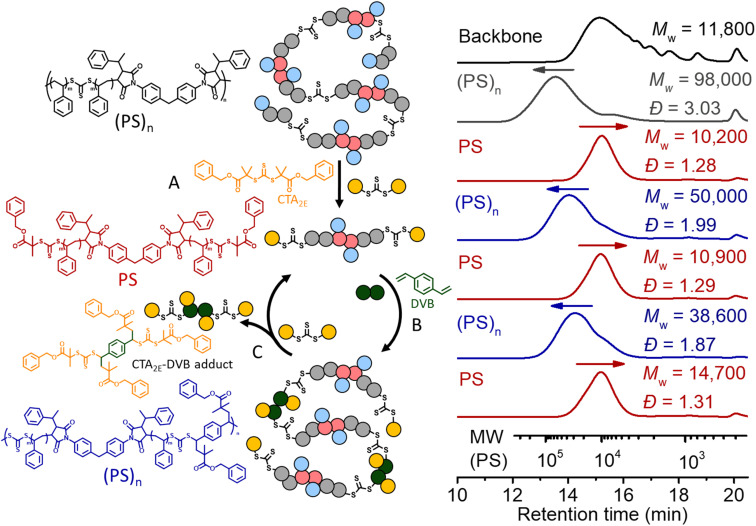
Recycling PS through RAFT step-growth polymerization and deconstruction. Black line: the backbone; grey line: chain expansion with St by RAFT chain-growth under 405 nm light with [St]_0_ : [TTC]_0_ = 500 : 1, conv.% = 14.1%; red line: deconstruction of the obtained polymer under 405 nm for 24 h with [TTC]_0_ : [CTA_2_]_0_ = 1 : 10; blue line: repolymerization of PS under 405 nm for 24 h with [PS]_0_ : [DVB]_0_ = 1 : 1.

Fig. 10, deconstruction of the (PS)_*n*_ (grey line, *M*_w_ = 98 000, *Đ* = 3.03) with CTA_2E_ resulted in PS with lower molecular weight and narrow MWD (*M*_w_ = 10 200, *Đ* = 1.28), indicating the high efficacy of CTA_2E_ for the deconstruction (step A). Divinylbenzene (DVB) was then chosen as the bifunctional vinyl monomer for RAFT step-growth (re)polymerization of these PS macro-CTAs (step B), because of the highly efficient RAFT-SUMI process between the styrene monomer and trithiocarbonate bearing tertiary alkyl carboxyl fragmentation (Fig. S20 and S21†). Successful repolymerization with DVB was observed by the shift of the SEC curve towards higher molecular weight distribution (*M*_w_ = 50 000, *Đ* = 1.99). Similar to the case of recycling PMA, the adduct of DVB and CTA_2E_ was generated during the RAFT interchange for the second deconstruction (step C); however, unlike the case of recycling PMA, this adduct can be easily removed by precipitation in methanol whilst retaining the PS macro-CTA (*M*_w_ = 10 900, *Đ* = 1.29). With the purified PS macro-CTA, we conducted the second repolymerization and third deconstruction through the same methods. The yield of PS obtained after each deconstruction and repolymerization is around 93–98%, showing that it is a promising method towards circular recycling of PS. The thermal decomposition temperature of the multiblock polystyrene was assessed by thermogravimetric analyses (TGA). The thermal decomposition temperature at 5% mass loss (*T*_d_, 5%) was slightly lower (371.6 °C) than that of non-degradable high molecular weight PS (reported value of 408 °C) (Fig. S22†).^7^ In theory, the thermal decomposition of the trithiocarbonate core is expected to occur between 210 and 250 °C;^68^ however, such transition is not observable due to the relatively low abundance of the trithiocarbonate by weight (∼1.52 wt%). Furthermore, Differential Scanning Calorimetry (DSC) revealed a glass transition temperature (*T*_g_) of 106.7 °C which matched well with expected values for high molecular weight PS (Fig. S23†).^7^ Furthermore, thermomechanical properties were investigated with temperature sweep experiments of Dynamic Mechanical Analysis (DMA); at temperatures below the *T*_g_, the elastic modulus was found to be within a similar order of magnitude to that of commercial PS (Fig. S24†).^69^

## Conclusions

In summary, RAFT step-growth polymerization *via* the Z-group approach was developed with a symmetrical trithiocarbonate as the CTA_2_ and a bismaleimide as the M_2_, resulting in an easy and efficient preparation of poly(trithiocarbonate)s. The in-chain dormant trithiocarbonate CTAs can be further utilized for chain expansion and as dynamic covalent bonds for deconstruction. Degradable PMA, PS and their multiblock block copolymers were successfully prepared by one step or two step chain expansion under 405 nm light. Segments generated after deconstruction showed controlled molecular weights and narrow molecular weight distributions. Moreover, these segments can be further repolymerized through the Z-group approach. We anticipate further expansion of the monomer scope for this new archetype of RAFT step-growth polymerization, considering many monomer and CTA pairings have already been successfully demonstrated through the former R-group approach. This will subsequently allow greater diversity of the step-growth backbone containing the in-chain CTAs and greater selection of vinyl monomers for chain-expansion. Thus, this new polymerization methodology can be considered as a general platform for preparing both degradable and recyclable yet functional vinyl polymers.

## Data availability

All experimental data is available in the ESI.†

## Author contributions

The manuscript was written through contributions of all authors.

## Conflicts of interest

The authors declare no conflict of interest.

## Supplementary Material

SC-015-D3SC06736J-s001
