# Supplementary material for: RAFT step-growth polymerization via the Z-group approach and deconstruction by RAFT interchange
Source: Chem Sci. 2024 Feb 23;15(13):4910–9. doi: 10.1039/d3sc06736j (PMC10966970; doi:10.1039/d3sc06736j)
Supplement: SC-015-D3SC06736J-s001 [file SC-015-D3SC06736J-s001.pdf]

Electronic Supplementary Material (ESI) for Chemical Science.
This journal is © The Royal Society of Chemistry 2024

Supporting Information For

RAFT Step Growth Polymerization via the Z-Group Approach and Deconstruction by RAFT interchange

Jiajia Li,[a,b] Joji Tanaka,[*b] Qing Li,[a] Claire Jing Jing Wang,[b] Sergei Sheiko,[b] Samantha Maria Clouthier,[b] Jian Zhu[a] and Wei You[*b]

[a] State and Local Joint Engineering Laboratory for Novel Functional Polymeric Materials, Jiangsu Key Laboratory of Advanced Functional Polymer Design and Application, Suzhou key Laboratory of Macromolecular Design and Precision Synthesis, Department of Polymer Science and Engineering, College of Chemistry, Chemical Engineering and Materials Science, Soochow University, Suzhou, 215123, China.

[b] Department of Chemistry, University of North Carolina at Chapel Hill, Chapel Hill, NC, 27599-3290, USA. E-mail: wyou@unc.edu

Experimental Section

General considerations. Unless stated all reagents were purchased from commercial suppliers and used as received. Dioxane was passed through activated basic aluminium oxide and discarded after several uses. For kinetic analysis A 22-gauge, 4 inch hypodermic needle (air-tite product) was used to sample the reaction mixture under argon flow. Typically, a single needle and syringe was used to take sample for both THF-SEC and ^{1}H NMR spectroscopy, by drawing out approximately 50 to 100 μL and dropping a few drops into a vial for ^{1}H NMR and the remaining mixture left in the needle was flushed out with THF (or vice versa) in a separate vial for SEC analysis.

Theoretical average molecular weight. The evolution of number-average (M_n), weight-average (M_w) and Z-average (M_z) molecular weight of the crude reaction mixture were determined by SEC analysis. These were plotted together with conversion determined by ^{1}H NMR with expected molecular averages for linear step-growth polymerization according to Flory's equation[1] which assumes balanced stoichiometry and no cyclization:

$$M_{n,\,th} = M_0 \frac{1}{1-p}$$

$$M_{w,\,th} = M_0 \frac{1+p}{1-p}$$

$$M_{z,\,th} = M_0 \frac{1+4p+p^2}{1-p^2}$$

Theoretical average molecular weight by approximating imbalanced stoichiometry from initiation was given according to literature[2] by replacing p with $r^{1/2}p$:

$$r_{\mathrm{th}} = \frac{1}{\frac{1}{r_0} + 4f\frac{[\mathrm{I}]_0}{[\mathrm{M}]_0}\left(1 - e^{-k_d t}\right)}$$

$$M_{w,th} \approx M_0 \frac{1 + {r_{th}}^{1/2} p}{1 - {r_{th}}^{1/2} p}$$

M_0 is the molecular weight of average molecular weight of **CTA₂** and **M₂** comonomers. r_0 is ratio of CTA to monomer (r_0 = $[CTA]_0/[M]_0$).

Limitations: It is important note, the initiation efficiency, f is expected fall particularly at high monomer conversion, therefore when the reaction is left for longer period after the polymerization reaches high monomer conversion, therefore resulting in overestimation of imbalanced stoichiometry. In particular, the shortcoming of constant value of f (0.65) is evident when the predicted $M_{w,th}$ falls as the reaction proceeds instead of increasing with conversion. To account for this, we recommend using r_{th} values calculated at earlier time points, assuming initiation efficiency falls at high conversion.

Instrumentation. All NMR spectra reported were recorded on a Bruker 400 MHz spectrometer in $CDCl_3$. All NMR spectra were processed using Mestrenova 11.0. Conventional SEC analysis of the crude polymerization mixture was carried out using polystyrene calibration in THF with calibration range of molecular weight from 195k to 370. These were measured using Waters 2695 separations module liquid chromatograph equipped with two Agilent ResiPore columns (linear SEC separation range up to 500 k) maintained at 35 °C, and a Waters 2412 refractive index detector. THF (without additives) was used as the mobile phase and the flow rate

was set to 1 mL/min. All the samples were run with 100 μL injection volume. The dRI response was measured from the start of the peak (at low retention time) down to the high retention time corresponding to the initial species (M_0) at t = 0, as presented in the image below.

Mark-Houwink analysis and absolute molecular weight analysis were carried out using multidetector detector GPC system (RI, LS, VS). These were measured using Agilent 1260 infinity Series (Degasser, Isocratic pump, Autosampler) and Wyatt Technology Corporation detectors (Dawn Hellos – II 18 angle MALS detector, Viscostar-II viscometer, Optilab T-rEX differential refractive index detector) equipped with 3 x PLGel-Mixed-BLS (linear SEC separation range of molecular weight from 10 M to 500) and 10 μm PLgel guard column. THF (with BHT stabilizer) was used as the mobile phase and the flow rate was set to 1 mL/min. All the samples were run with 100 μL injection volume. dn/dC of the polymer samples in THF were determined by the instrument assuming 100 % mass recovery.

Thermogravimetric analyses (TGA) were measured under nitrogen with TGA-Q500 from TA instruments. Approximately 5 mg of sample was heated at 10 °C/min from 25 °C to 800 °C.

Differential Scanning Calorimetry (DSC) thermograms were obtained with DSC-Q100 from TA instruments. Approximately 5 mg of the sample was measured and added to an aluminum sealed pan that passed through a heating/cooling cycle at 10 °C/min. Reported data are from the third full cycle. The temperature ranged from -10 °C to 150 °C.

Dynamic mechanical analysis (DMA) samples were cast from xylene with concentration of ~20% in dumbbell-shaped molds and left to dry overnight at room temperature then at 75 °C for 72 hrs. The dumbbell samples of dimensions of 9 mm × 2 mm × 1.55 mm were used for mechanical analysis using an RSA-G2 dynamic mechanical analysis system from TA Instruments. Temperature sweeps were performed at a frequency of 1 Hz and an amplitude of 0.01% strain from 25-150 °C at a 3 °C temperature step and a soaking time of 120 s per temperature point.

All polymerizations were carried out in 3.7 ml scintillation vials with a diameter of 15 mm, or in 20 ml scintillation vials with a diameter of 28 mm. The 405 nm LED lamp (200W, 60 mW/cm^{-2}) was purchased from FASTTOBUY. The vails was put in front of the lamp at a distance of around 5 cm.

Synthesis of CTA_2: CTA_2 was synthesized according to a slightly revised procedure from literature.[3] Cs_2CO_3 (16 g, 50 mmol) was added to ACN (50 mL) and stirred for 1 hour at 25 °C. Then CS_2 (4.6 g, 60 mmol) was added and stirred for 0.5 hour. After that, (1-bromoethyl) benzene (8.3 g, 45 mmol) was added for further reaction time of 40 hours. The reaction was washed by 100 mL water and extracted by 100 mL EtOAc three times. The organic phase was dried over Na_2SO_4 and evaporated to dryness to give pure product as an orange oil (6.86 g, 21.6 mmol, 96.0%).

RAFT SUMI model reaction between CTA_2 and PPD: A mixture of *N*-phenylmaleimide (**PPD**, 0.27 g, 1.6 mmol), and **CTA_2** (0.3 g, 0.8 mmol) and AIBN (13 mg, 80 μmol) was placed in a 4 mL scintillation vial. TCE (1.6 mL) was added as solvent and two drops of DMF from the syringe needle was added as internal standard for ^1H NMR analysis. The content was deoxygenated by purging argon for 10 min prior to placing under heat at 70 °C. In predetermined intervals, a small amount of reaction solution was extracted with a needle for analysis. The ^1H NMR samples were taken under positive argon pressure. The monomer conversion was determined by integrating the vinyl-proton at 6.88 ppm. The SUMI-CTA adduct yield was determined by integrating the CH peak next to the trithiocarbonate at 4.45–4.95 ppm. The CTA consumption was calculated by integrating the vinyl-proton at 5.26 ppm.

RAFT Step growth polymerization. M_2 (180 mg, 0.49 mmol), **CTA_2** (160 mg, 0.49 mmol), and AIBN (8 mg, 0.05 mmol) was added into the vial, followed by 979 μL of TCE, targeting a molar concentration of $[\mathbf{M_2}]_0$:$[\mathbf{CTA_2}]_0$:$[AIBN]_0$ = 0.5 : 0.5 : 0.05 M. Two drops of DMF from the syringe

needle was added as internal standard for ^{1}H NMR analysis. The solution was sealed with a rubber septum, degassed with argon for 10 minutes, and then placed on a heat block at 70 °C for 4 hours to react. After polymerization, the solution was diluted by DCM and precipitated in ethyl ether. The monomer conversion was determined by integrating the vinyl-proton at 6.88 ppm. Molecular weight and molecular weight distribution were determined by SEC.

Chain expansion of the backbone. The chain expansion of backbone with MA was described as the typical example. A mixture of MA (1.6 g, 18.6 mmol), backbone polymer (25 mg, 3.4 mmol) was dissolved in dioxane (1 mL) and placed in a 4 mL scintillation vial with the molar ratio of $[MA]_0$:[$\mathbf{CTA_2}$]$_0$ = 500:1. Two drops of DMF from the syringe needle was added as internal standard for ^{1}H NMR analysis. The content was degassed by purging argon for 10 min prior to placing in front of a 405 nm UV lamp. After the desired reaction time at room temperature, the vial was exposed to oxygen and the solution was diluted by THF. The polymer was obtained after precipitation in hexane and then dried under vacuum at 40 °C. The monomer conversion was determined by integrating the vinyl-proton at 5.83 ppm. Molecular weight and molecular weight distribution were determined by SEC.

Preparation of the multiblock copolymer. The chain expansion of backbone with St was first conducted according to the procedure mentioned above with the molar ratio of $[St]_0$:[$\mathbf{CTA_2}$]$_0$ = 330:1. The PS was obtained after precipitation in methanol. Then the purified PS was used as macroRAFT agent for the second chain expansion with $[MA]_0:[PS]_0$ = 330:1. The multiblock copolymer was obtained after precipitation in hexane. The monomer conversion of St was determined by integrating the vinyl-proton at 5.81 ppm. The monomer conversion of MA was determined by integrating the vinyl-proton at 5.83 ppm. Molecular weight and molecular weight distribution were determined by SEC.

Deconstruction of the backbone. A mixture of backbone (0.2 g, 38.4 μmol), $\mathbf{CTA_2}$ (0.47 g, 1.47 mmol) with the molar ratio of [**TTC**]$_0$:[$\mathbf{CTA_2}$]$_0$ =1:5 was dissolved in dioxane (1 mL) and placed in a 4 mL scintillation vial. The content was degassed by purging argon for 10 min. The reaction was conducted at room temperature under 405 nm LED for 2 hours. The solution was diluted by THF prior to precipitation in diethyl ether. The product was obtained after drying under vacuum at 40 °C. Molecular weight and molecular weight distribution were determined by SEC.

Repolymerization of the mixture after deconstruction in Figure 6. The repolymerization was conducted by adding $\mathbf{M_2}$ to the mixture after deconstruction with the molar ratio of [$\mathbf{M_2}$]$_0$:[$\mathbf{CTA_2}$]$_0$ =1:1 at room temperature under 405 nm LED for 72 hours. The solution was diluted by THF prior to precipitation in diethyl ether. The product was obtained after drying under vacuum at 40 °C. Molecular weight and molecular weight distribution were determined by SEC.

Deconstruction of PMA with $\mathbf{CTA_2}$. Deconstruction of PMA (M_w = 140,100, $Đ$ = 2.99) was described as the typical example. The number of **TTC** (n = 11) was determined from the backbone before chain expansion. A mixture of PMA (0.1 g, 2.12 μmol), $\mathbf{CTA_2}$ (0.074 g, 0.23 mmol) with the molar ratio of [**TTC**]$_0$:[$\mathbf{CTA_2}$]$_0$ =1:10 was dissolved in dioxane (1 mL) and placed in a 4 mL scintillation vial. The content was degassed by purging argon for 10 min. The reaction was conducted at room temperature under 405 nm LED for 24 hours. The solution was diluted by THF prior to precipitation in hexane. The product was obtained after drying under vacuum at 40 °C. Molecular weight and molecular weight distribution were determined by SEC.

Repolymerization of PMA. Repolymerization of PMA (M_w = 1,800, $Đ$ = 1.14) was described as the typical example. PMA (0.3 g, 0.19 mmol) and $\mathbf{M_2}$ (0.068 g, 0.19 mmol) was first dissolved in 0.4 mL TCE. The content was degassed by purging argon for 10 min. The reaction was conducted at room temperature under 405 nm LED for 96 hours. The solution was diluted by THF prior to precipitation in hexane. The product was obtained after drying under vacuum at 40 °C. Molecular weight and molecular weight distribution were determined by SEC.

RAFT-SUMI model reaction between BTMPA and Styrene. AIBN (15.8 mg, 0.096 mmol), Styrene (200 mg, 1.92 mmol), 2-(((butylthio)carbonothioyl)thio)-2-methylpropanoic acid (**BTMPA**, 484.7 mg, 1.92 mmol) and dioxane (1.7 ml) was charged into a sealed scintillation vial. The

mixture was deoxygenated by purging with argon for 10 minutes prior to heating at 70 °C for 4 hours. Monomer consumption, CTA consumption and RAFT-SUMI yield was determined from ^{1}H-NMR relative to CH_3 on the Z-group. Monomer consumption was measured from the vinyl peak at 5.26 ppm and RAFT-SUMI yield was measured from CH peak next to the trithiocarbonate at 5.37 ppm. CTA consumption was approximated from peak fitting a region belonging to CH_2 of the initial CTA and SUMI adduct at 3.31-3.33 ppm by assuming integral ratio of triplet to be 1:2:1.

Synthesis of CTA_{2E}: 2,2'-(thiocarbonylbis(sulfanediyl))bis(2-methylpropanoic acid) (10 g, 35.5 mmol, 1 eq) was suspended in 100 ml DCM in RBF equipped with stir bar. Whilst stirring under ice bath, 1,8-Diazabicyclo[5.4.0]undec-7-ene (11.88 g, 78.0 mmol, 2.2 eq) was added, followed by benzyl bromide (13.3 g, 78.0 mmol, 2.2 eq) and left to stir over night at room temperature. The reaction mixture was directly subjected to separation by silica column chromatography (800 g, SiO_2) using 5 % ethyl acetate in hexanes as the eluent. The purified material was further subjected to recrystallization in ethanol to yield crystals (11.8 g, 72 %).

Deconstruction of PS with CTA_{2E}. Deconstruction of PS (M_w = 98,000, $Đ$ = 3.03) was described as the typical example. The number of **TTC** (n = 14) was determined from the backbone before chain expansion. A mixture of PS (0.15 g, 4.64 μmol), **CTA_{2E}** (0.3 g, 0.65 mmol) with the molar ratio of [**TTC**]$_0$:[**CTA_2**]$_0$ =1:10 was dissolved in dioxane (1.5 mL) and placed in a 4 mL scintillation vial. The content was degassed by purging argon for 10 min. The reaction was conducted at room temperature under 405 nm LED for 24 hours. The solution was diluted by THF prior to precipitation in methanol. The product was obtained after drying under vacuum at 40 °C. Molecular weight and molecular weight distribution were determined by SEC.

Repolymerization of PS. Repolymerization of PS (M_w = 10,200, $Đ$ = 1.28) was described as the typical example. PS (0.1221 g, 0.015 mmol) and **DVB** (1.99 mg, 0.015 mmol) was first dissolved in 0.4 mL dioxane. The content was degassed by purging argon for 10 min. The reaction was conducted at room temperature under 405 nm LED for 24 hours. The solution was diluted by THF prior to precipitation in methanol. The product was obtained after drying under vacuum at 40 °C. Molecular weight and molecular weight distribution were determined by SEC.

Additional Results

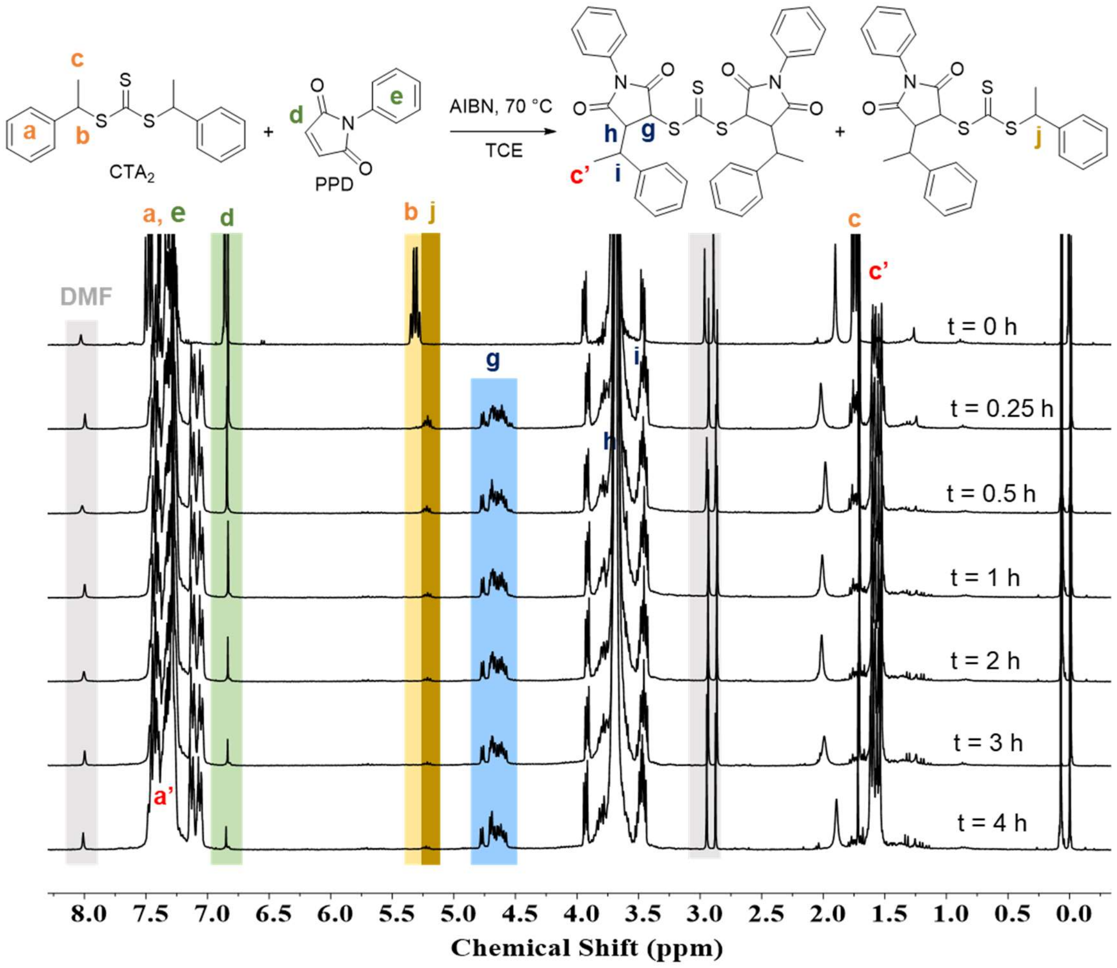

Figure S1. ^{1}H NMR ($CDCl_3$, 400 MHz) spectrum of RAFT-SUMI analysis of **CTA_2** and **M**. The yield of SUMI-**CTA_2** adduct was determined by proton g (blue). The CTA consumption was determined by proton b and j (yellow and brown). The monomer conversion was determined by proton d (green).

Table S1. RAFT-SUMI kinetics analysis of **CTA_2** and **M**.

Time (h)	$[M]_t/[M]_0$	$[CTA_2]_t/[CTA_2]_0$	SUMI-CTA_2 adduct yield
0	1	1	0
0.25	0.165	0.137	0.814
0.5	0.08	0.295	0.865
1	0.06	0.146	0.91
2	0.03	0.062	0.913
3	0.02	0.047	0.931
4	0.02	0.037	0.932

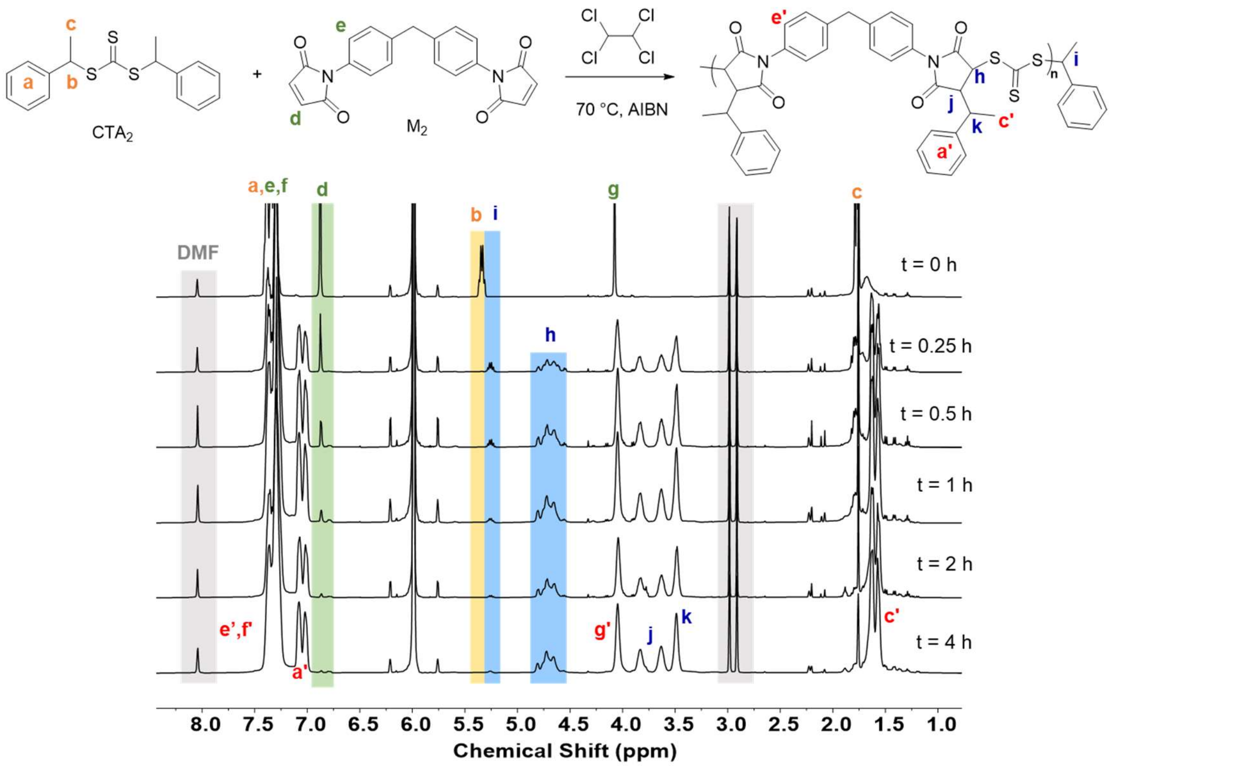

Figure S2. ^{1}H NMR ($CDCl_3$, 400 MHz) spectrum of RAFT step growth polymerization of $\mathbf{CTA_2}$ and $\mathbf{M_2}$ with $[\mathbf{CTA_2}]_0$:$[\mathbf{M_2}]_0$:$[AIBN]_0$ = 0.5:0.5:0.05 M in TCE at 70 °C. The monomer conversion was determined by proton d (green).

Table S2. Kinetic Data for polymerization between $\mathbf{CTA_2}$ and $\mathbf{M_2}$.

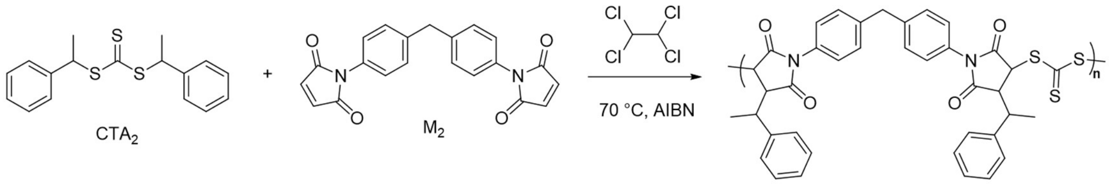

Entry	Time (h)	p	$M_{w, th}$	$M_{w, th}$ (r_{th})	M_w	M_w/M_n	M_z/M_w
1	0.25	0.82	3400	3400	2600	1.86	1.67
2	0.5	0.91	7200	6900	6700	2.04	1.66
3	1	0.96	16600	13800	13000	2.59	1.86
4	2	0.98	33500	19200	21500	3.30	2.18
5	4	0.99	67300	18600	32900	4.18	2.67

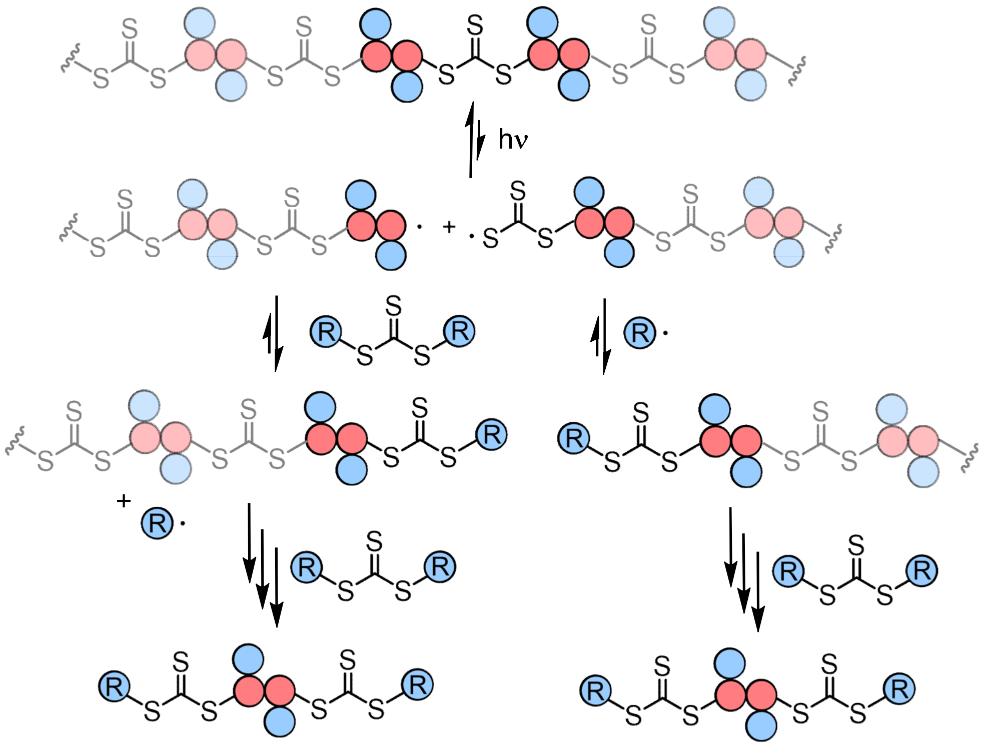

Figure S3. Proposed mechanism for the deconstruction of the backbone.

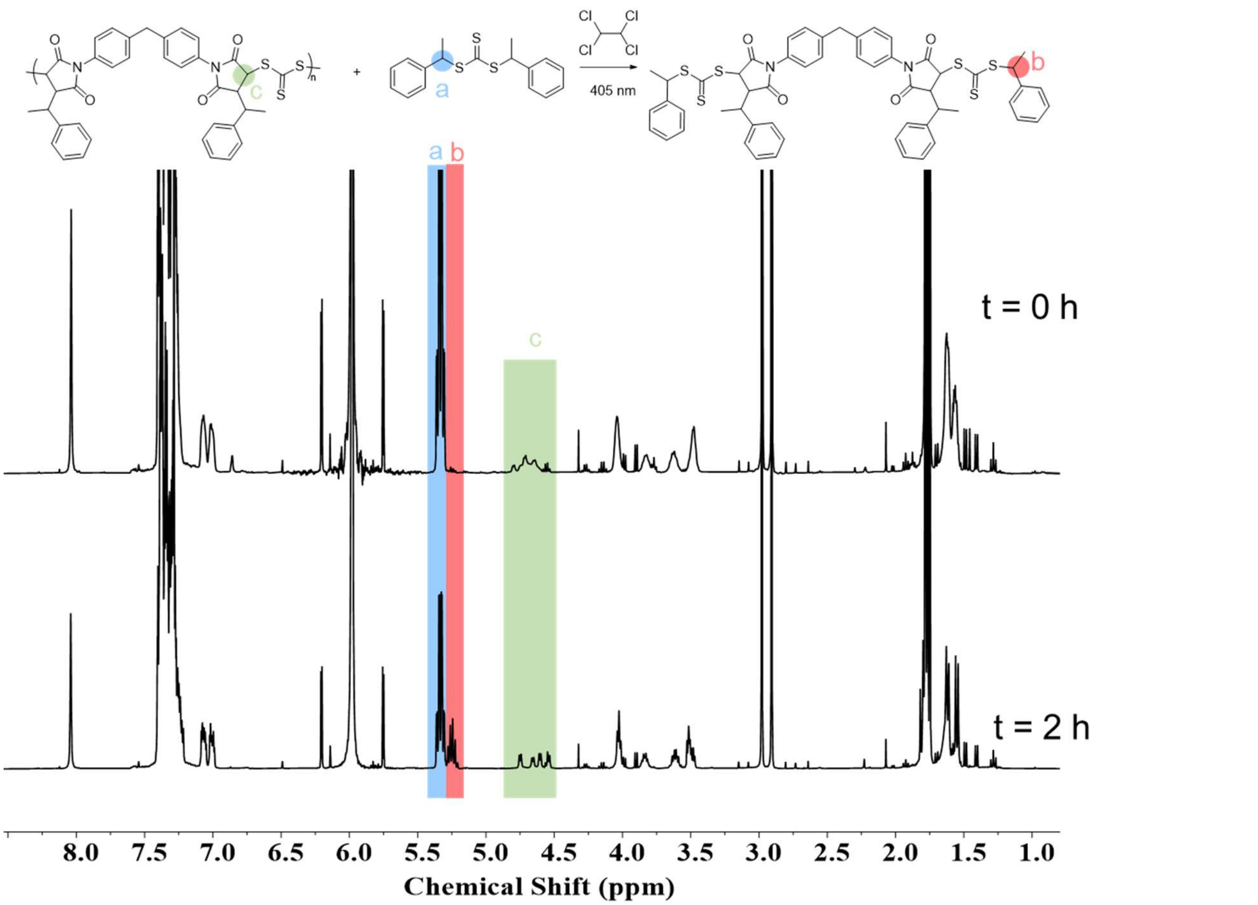

Figure S4. ^{1}H NMR ($CDCl_3$, 400 MHz) spectrum for deconstruction of the backbone with [**TTC**]$_0$:[**CTA$_2$**]$_0$ = 1:5 in TCE under 405 nm LED at 25 °C.

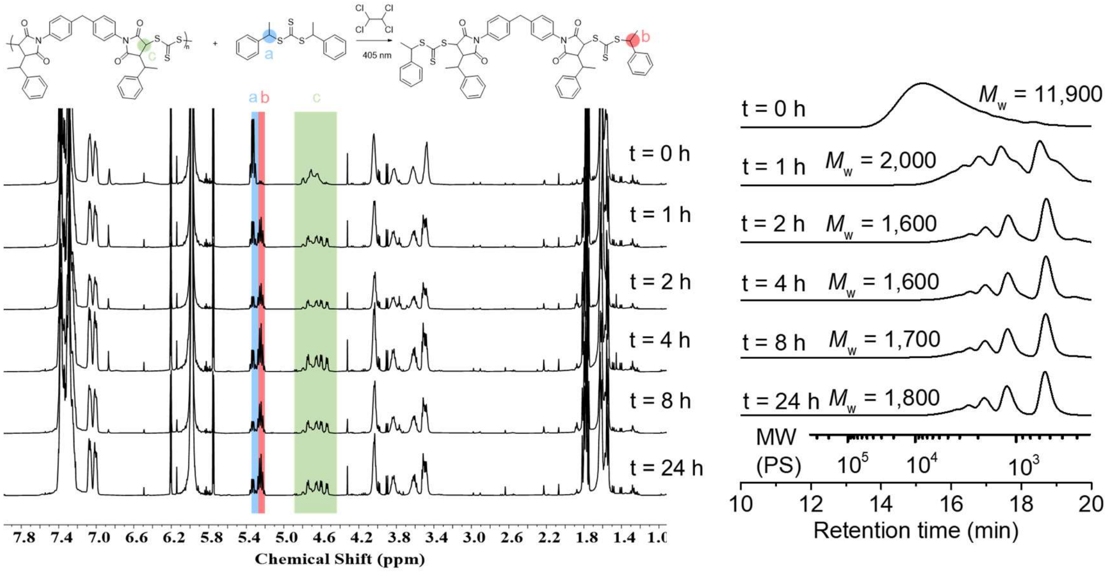

Figure S5. ^{1}H NMR ($CDCl_3$, 400 MHz) spectrum and SEC traces for deconstruction of the backbone with [**TTC**]$_0$:[**CTA$_2$**]$_0$ = 1:1 in TCE under 405 nm LED at 25 °C.

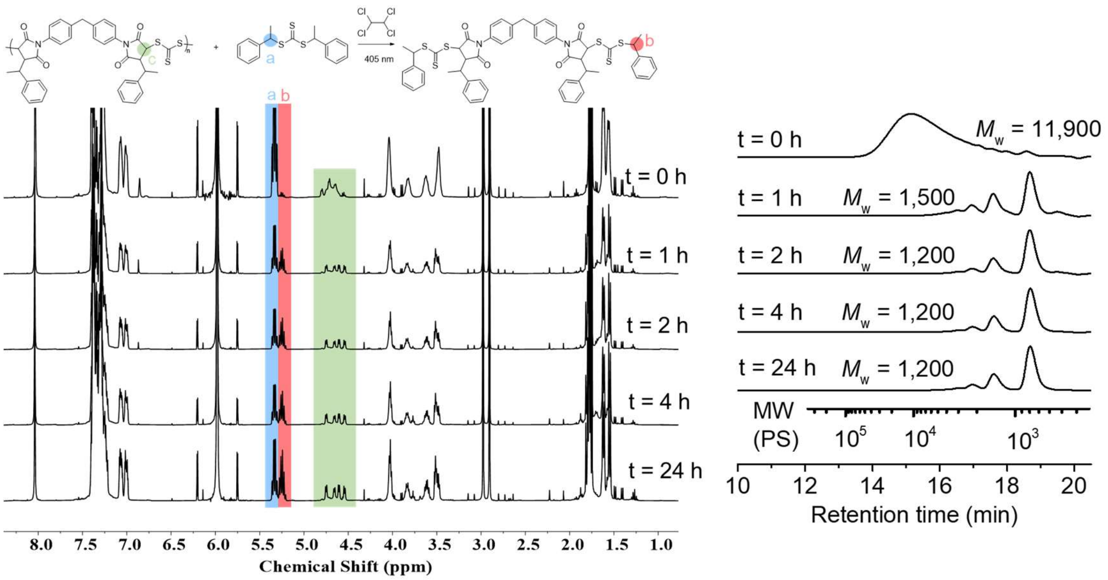

Figure S6. ^{1}H NMR ($CDCl_3$, 400 MHz) spectrum and SEC traces for deconstruction of the backbone with $[TTC]_0$:$[\mathbf{CTA_2}]_0$ = 1:2 in TCE under 405 nm LED at 25 °C.

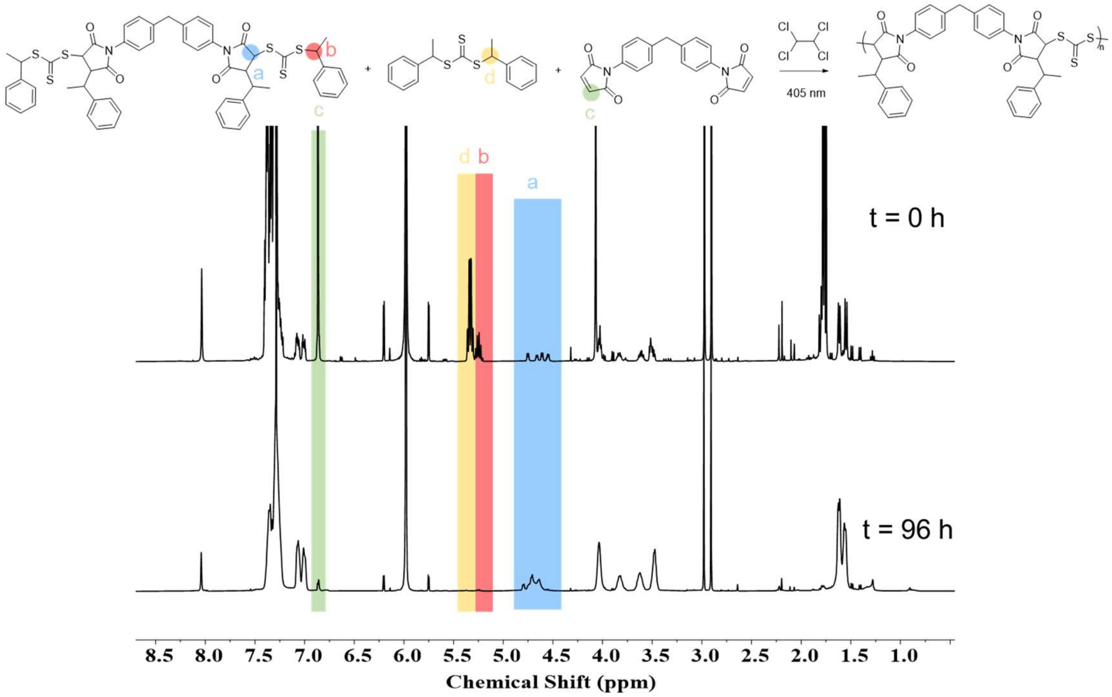

Figure S7. ^{1}H NMR ($CDCl_3$, 400 MHz) spectrum for repolymerization of the mixture after deconstruction of the backbone in **Figure 3** with $[\mathbf{M_2}]_0$:$[\mathbf{CTA_2}]_0$ = 0.5:0.5 M in TCE under 405 nm LED at 25 °C.

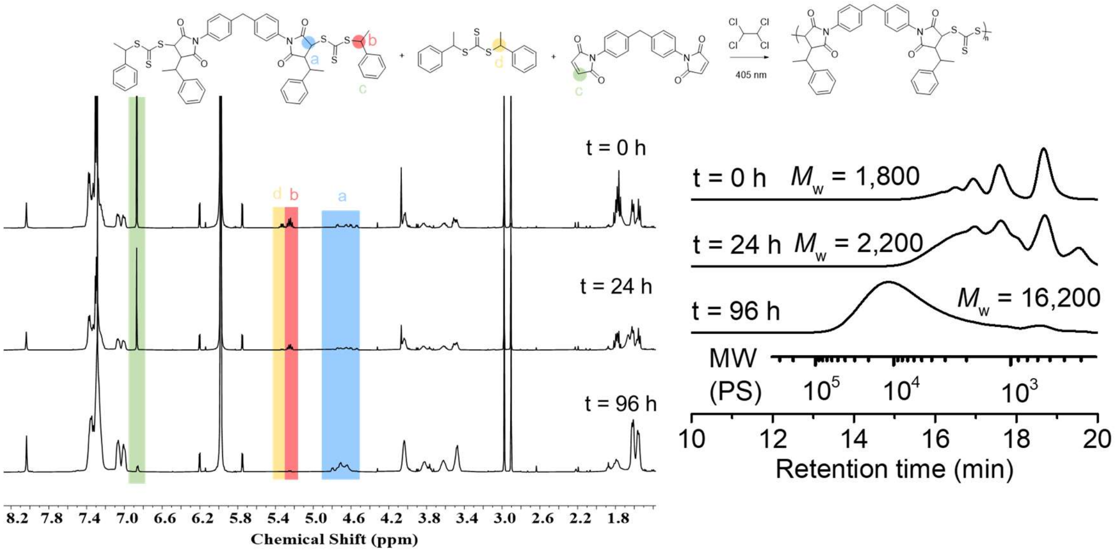

Figure S8. ^{1}H NMR ($CDCl_3$, 400 MHz) spectrum and SEC traces for repolymerization of the mixture after deconstruction of the backbone in **Figure S5** with [$\mathbf{M_2}$]$_0$:[$\mathbf{CTA_2}$]$_0$ = 0.125:0.125 M in TCE under 405 nm LED at 25 °C.

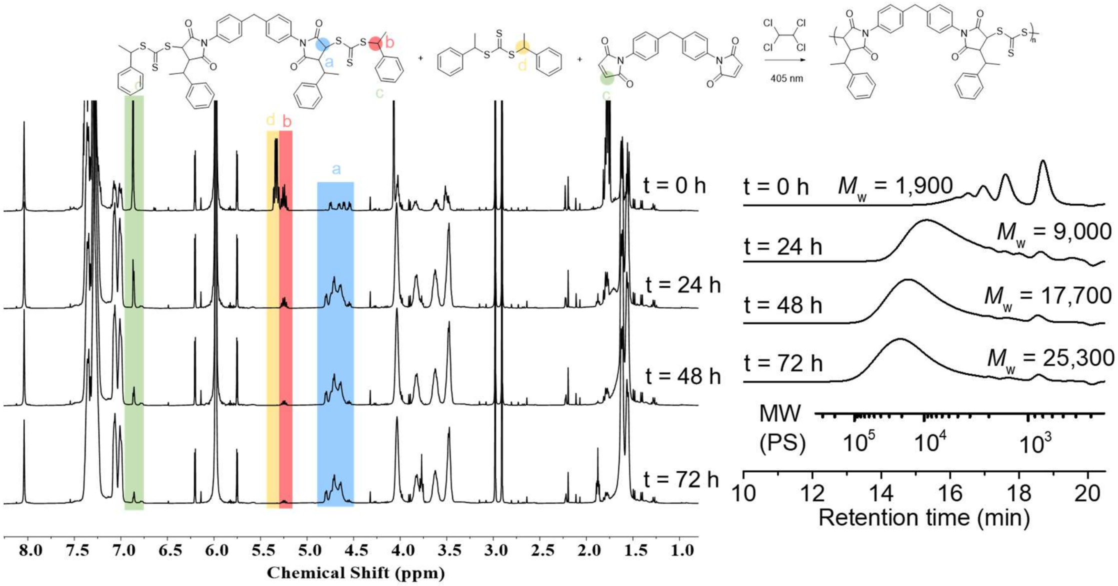

Figure S9. ^{1}H NMR ($CDCl_3$, 400 MHz) spectrum and SEC traces for repolymerization of the mixture after deconstruction of the backbone in **Figure S6** with [$\mathbf{M_2}$]$_0$:[$\mathbf{CTA_2}$]$_0$ = 0.5:0.5 M in TCE under 405 nm LED at 25 °C.

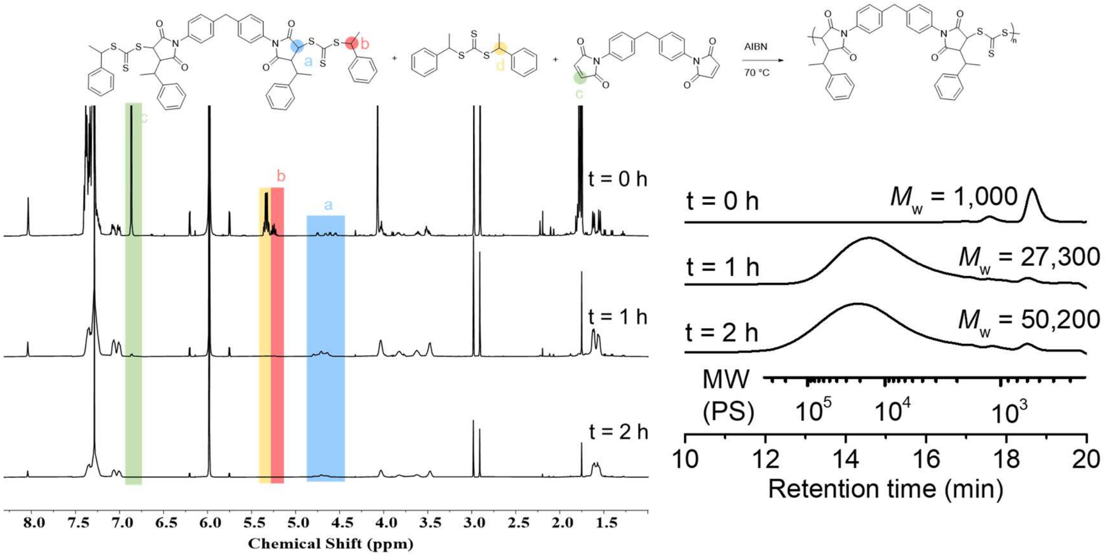

Figure S10. ^{1}H NMR ($CDCl_3$, 400 MHz) spectrum and SEC traces for repolymerization of the mixture after deconstruction of the backbone in **Figure 3** with [$\mathbf{M_2}$]$_0$:[$\mathbf{CTA_2}$]$_0$:[**AIBN**]$_0$ = 0.5:0.5:0.05 M in TCE at 70 °C.

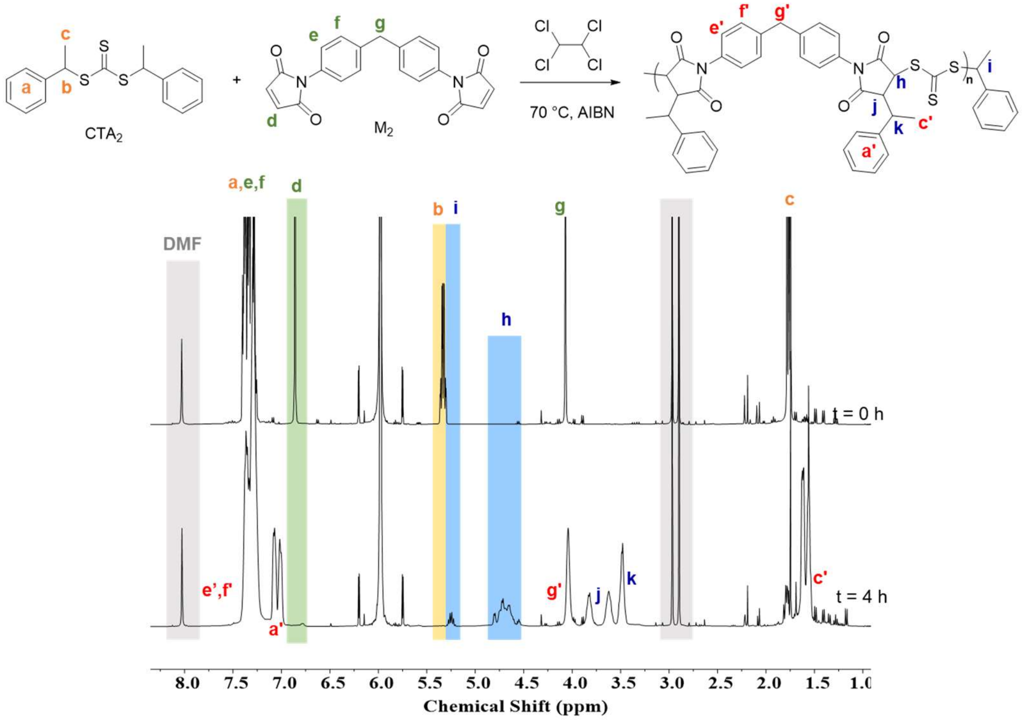

Figure S11. ^{1}H NMR ($CDCl_3$, 400 MHz) spectrum of RAFT step growth polymerization of $\mathbf{CTA_2}$ and $\mathbf{M_2}$ with [$\mathbf{CTA_2}$]$_0$:[$\mathbf{M_2}$]$_0$:[AIBN]$_0$ = 0.5:0.45:0.05 M in TCE at 70 °C.

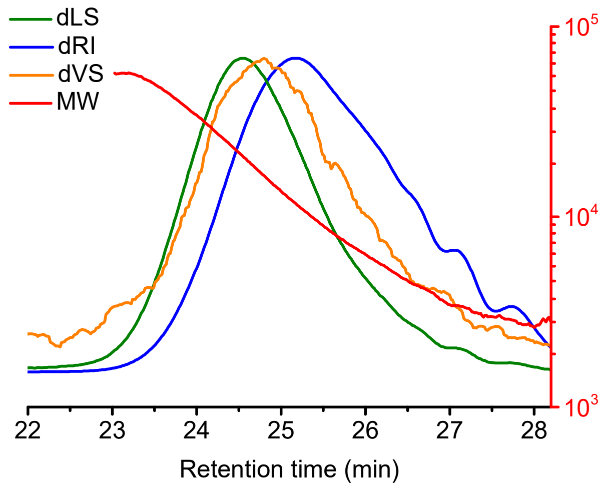

Figure S12. Molecular weight distributions of the backbone ($M_{w,LS}$ = 12.3 k, $M_{n,LS}$ = 7.2 k) obtained via triple-detection analysis.

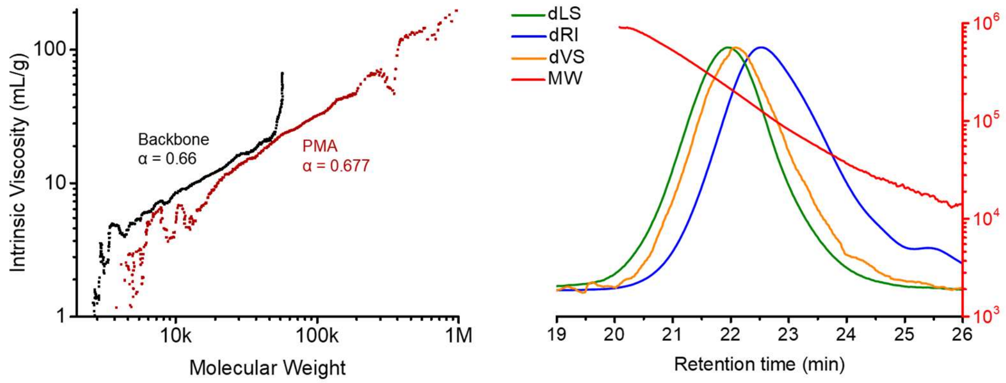

Figure S13. A) Mark-Houwink plots of the backbone (black) and PMA after chain expansion from **Figure 7A**; B) Molecular weight distributions of PMA ($M_{w,LS}$ =137 k, $M_{n,LS}$ = 59 k) obtained via triple-detection analysis.

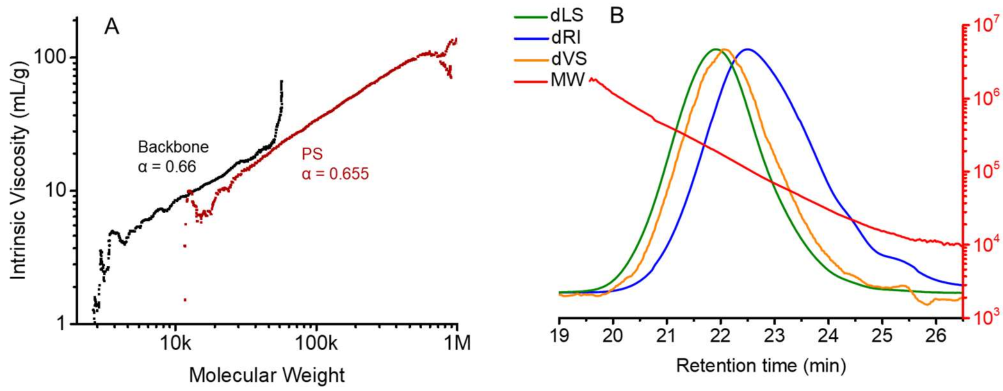

Figure S14. A) Mark-Houwink plots of the backbone (black) and PS after chain expansion from **Figure 7B**; B) Molecular weight distributions of PS ($M_{w,LS}$ = 119 k, $M_{n,LS}$ = 52 k) obtained via triple-detection analysis.

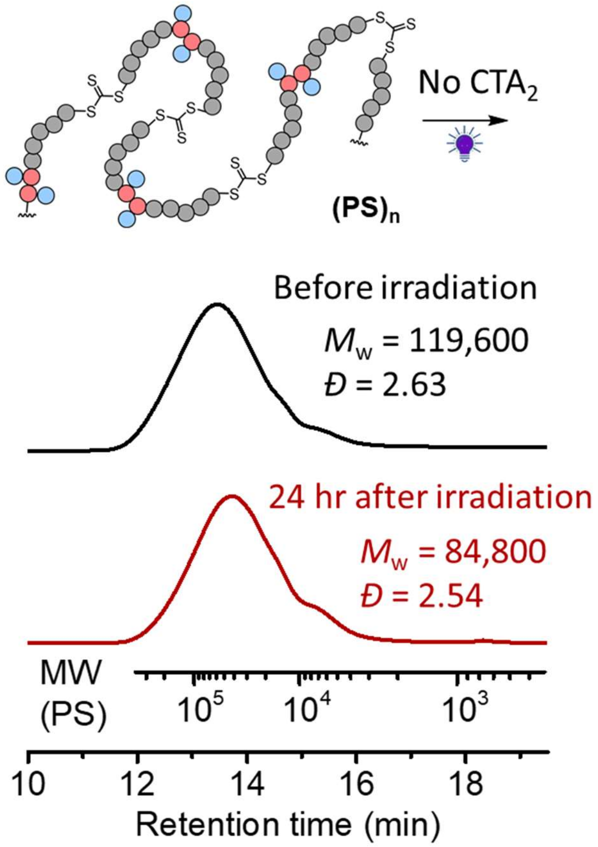

Figure S15. SEC analysis (THF, dRI) of (PS)n irradiated under the same reaction conditions in absence of CTA_2, revealing negligible effect in molecular weight distribution.

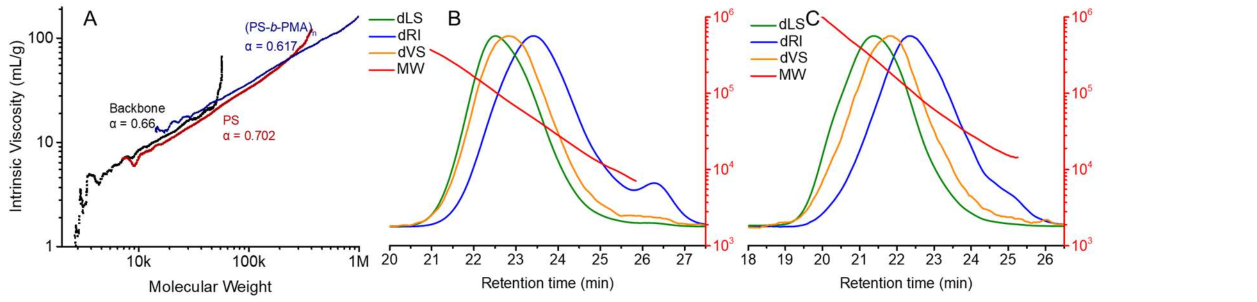

Figure S16. A) Mark-Houwink plots of the backbone, PS and (PS-*b*-PMA)$_n$ after chain expansion in **Figure 8**; B) Molecular weight distributions of PS ($M_{w,LS}$ = 58 k, $M_{n,LS}$ = 30 k) obtained via triple-detection analysis; C) Molecular weight distributions of (PS-*b*-PMA)$_n$ ($M_{w,LS}$ = 152 k, $M_{n,LS}$ = 63 k) obtained via triple-detection analysis.

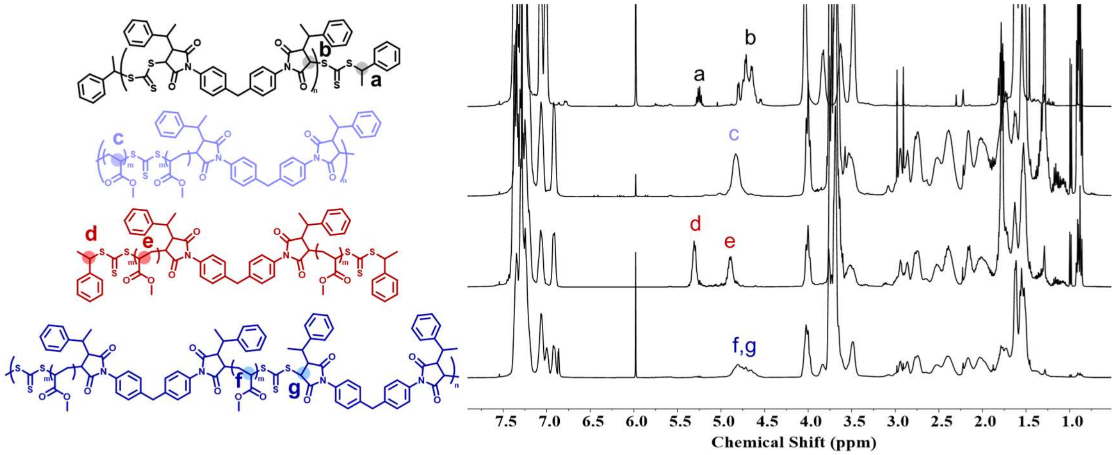

Figure S17. ^{1}H NMR spectra for recycling PMA in **Figure 9** in $CDCl_3$.

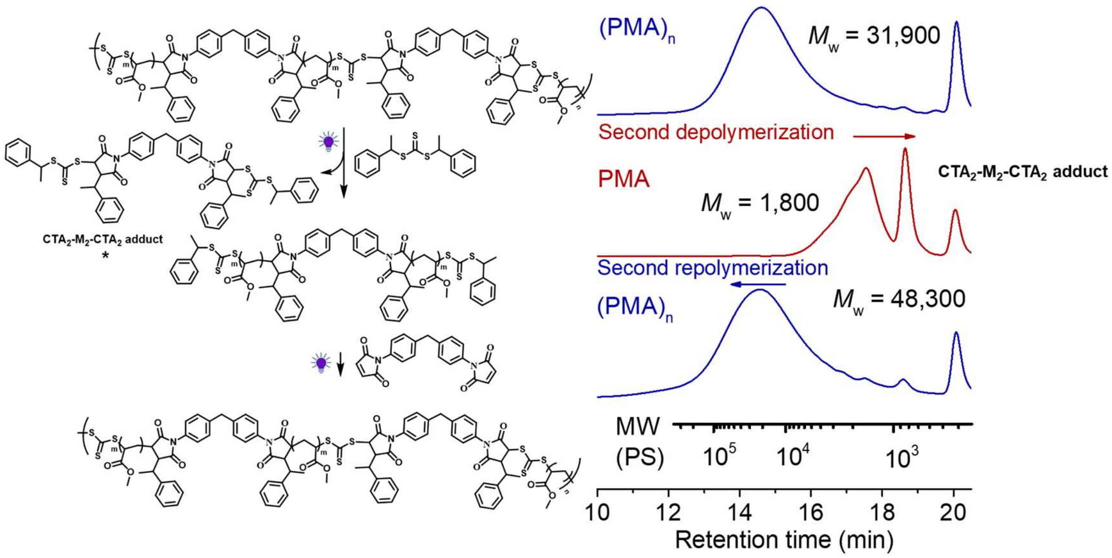

Figure S18. SEC traces for second deconstruction of PMA obtained in **Figure 9** with [**TTC**]$_0$:[**CTA$_2$**]$_0$ = 1:10 in dioxane under 405 nm LED at 25 °C for 24 h and second repolymerization of the PMA segments with [**PMA**]$_0$:[**M$_2$**]$_0$ = 1:1 in dioxane under 405 nm LED at 25 °C for 96 h.

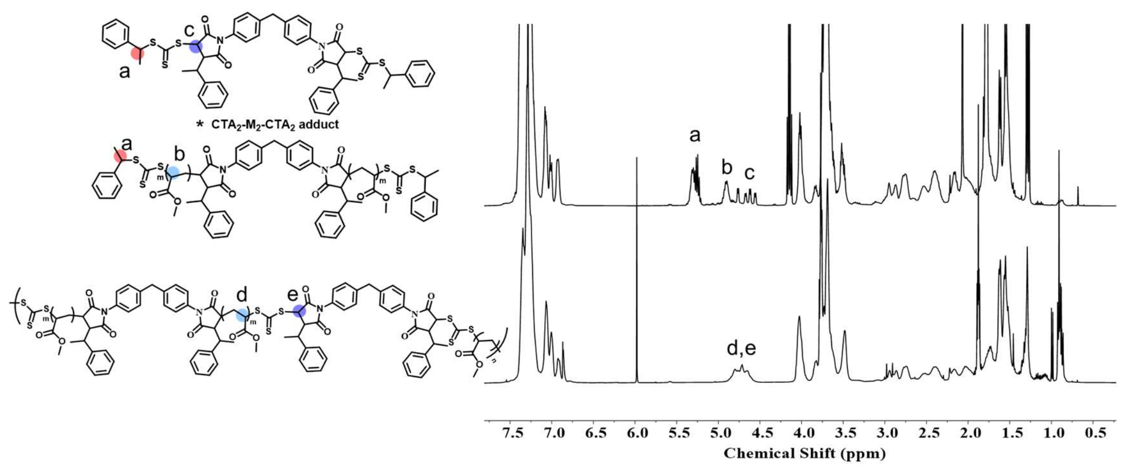

Figure S19. ^{1}H NMR spectra for second recycling of PMA in **Figure S18** in $CDCl_3$.

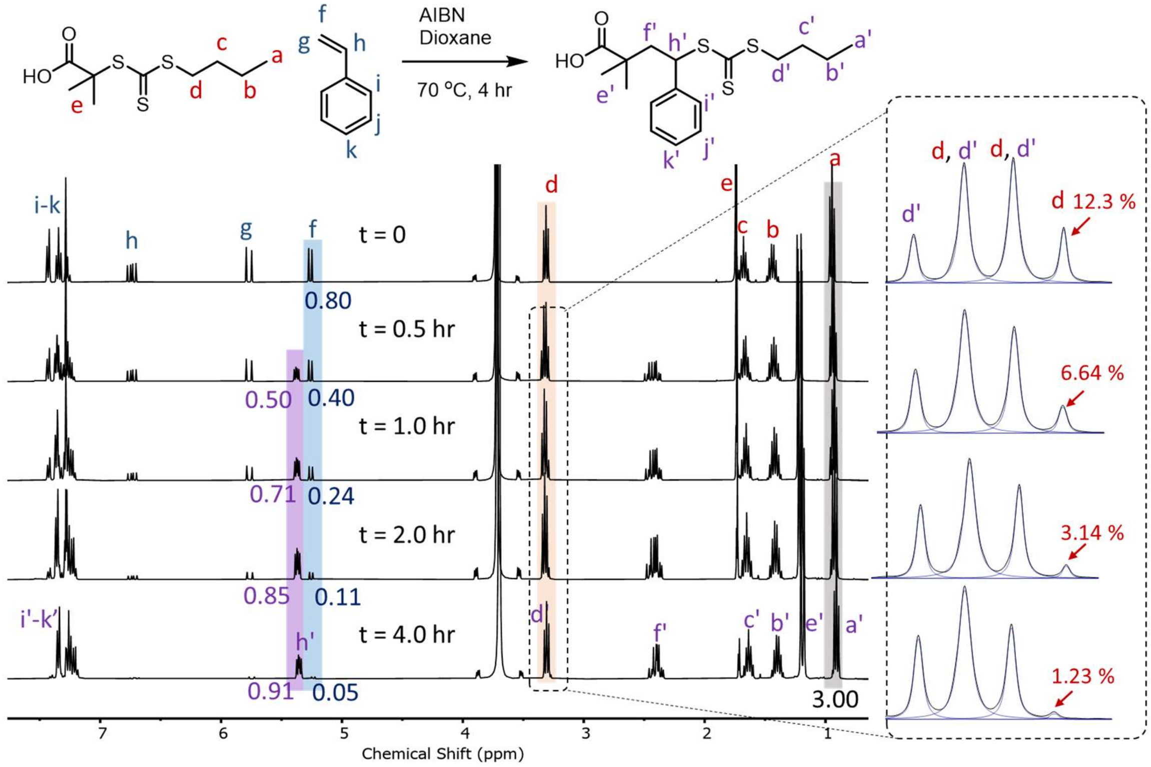

Figure S20. ^{1}H-NMR spectra of RAFT-SUMI of styrene with BTMPA.

Table S3: RAFT-SUMI of styrene with BTMPA.

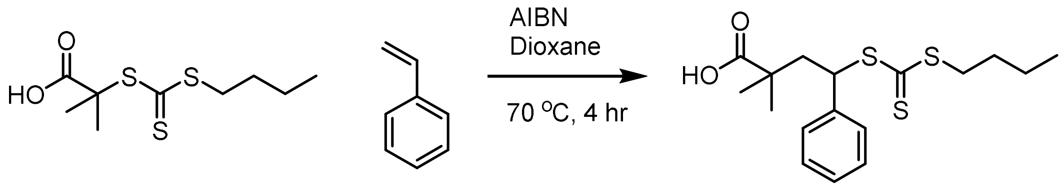

Time (hr)	$[M]_t/[M]_0$	$[CTA]_t/[CTA]_0$	SUMI-CTA yield
0	1.000	1.000	0.000
0.5	0.500	0.492	0.500
1.0	0.300	0.266	0.710
2.0	0.138	0.126	0.850
4.0	0.063	0.049	0.910

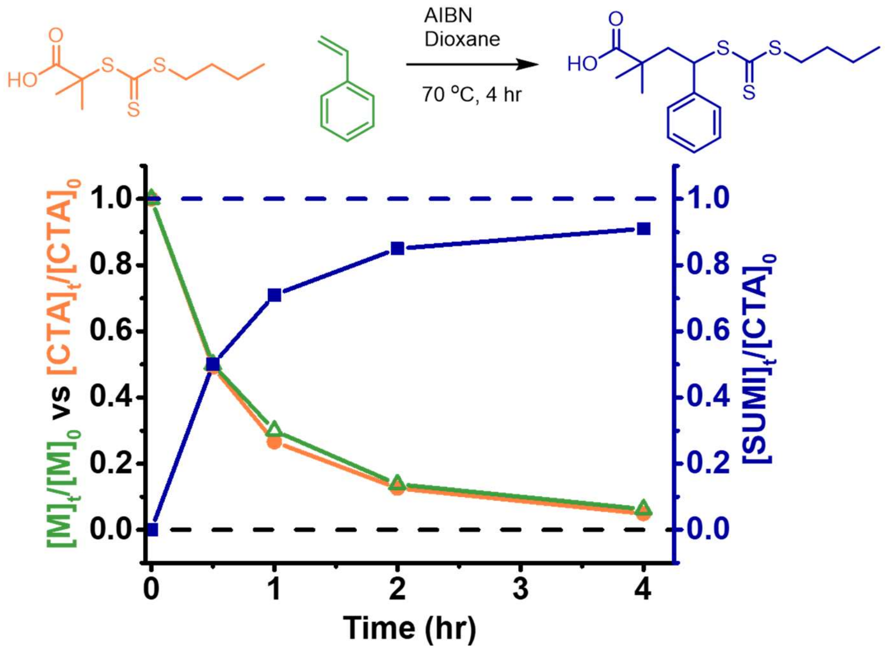

Figure S21. Kinetic plot of RAFT-SUMI of styrene with BTMPA.

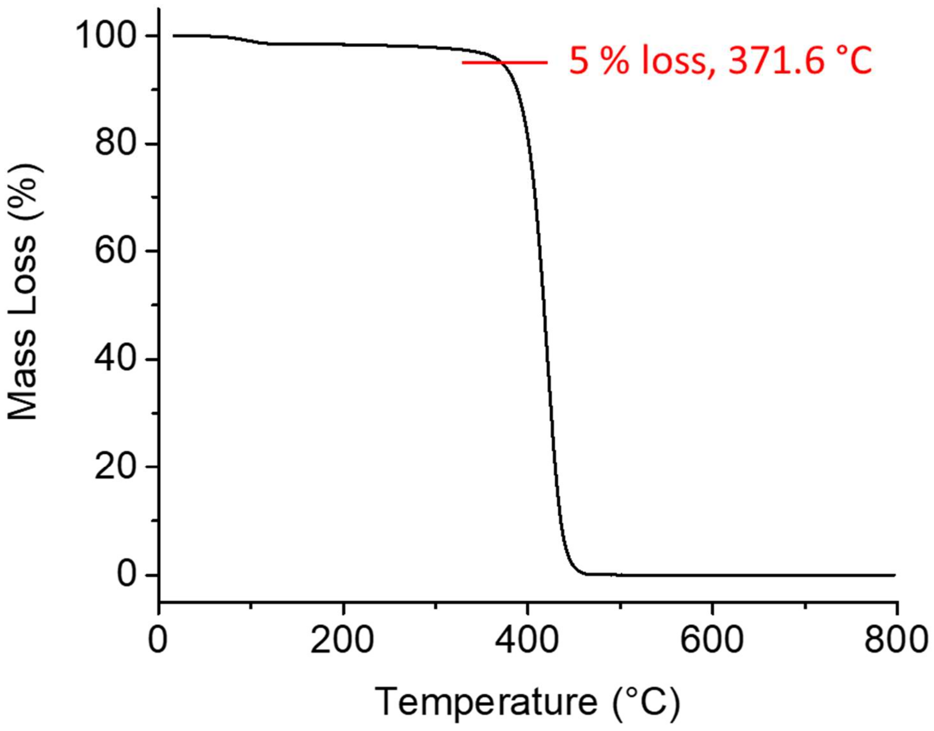

Figure S22. TGA thermogram of $(PS)_n$.

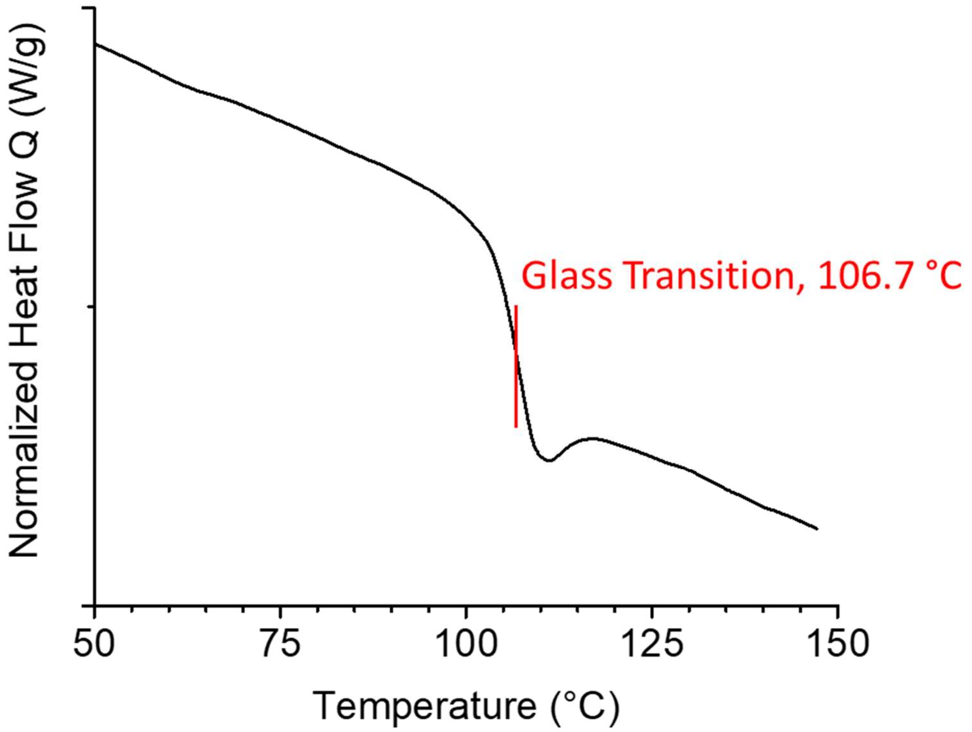

Figure S23. DSC thermogram of $(PS)_n$.

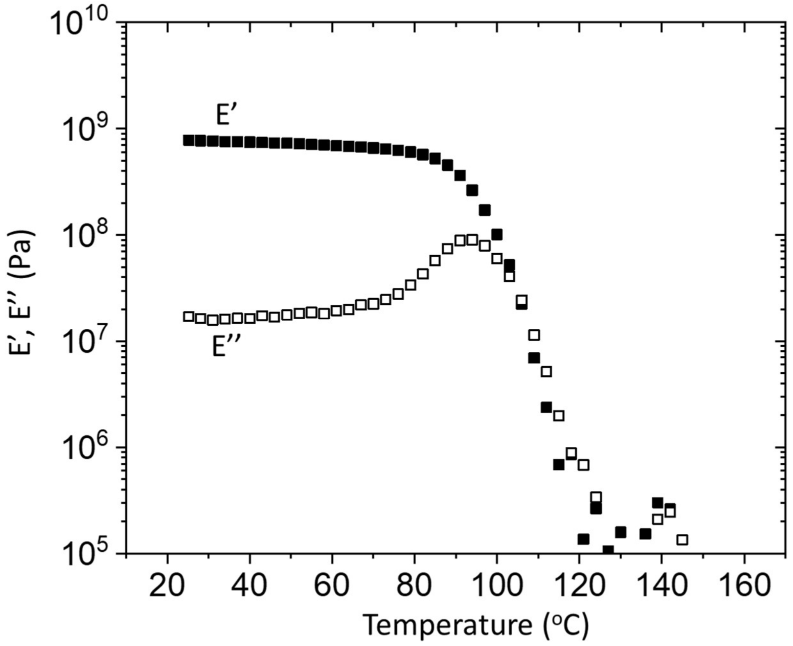

Figure S24. DMA temperature sweeps at constant amplitude (0.01 %) and frequency (1 Hz). E′ = elastic modulus (filled squares); E″ = loss modulus (unfilled squares).

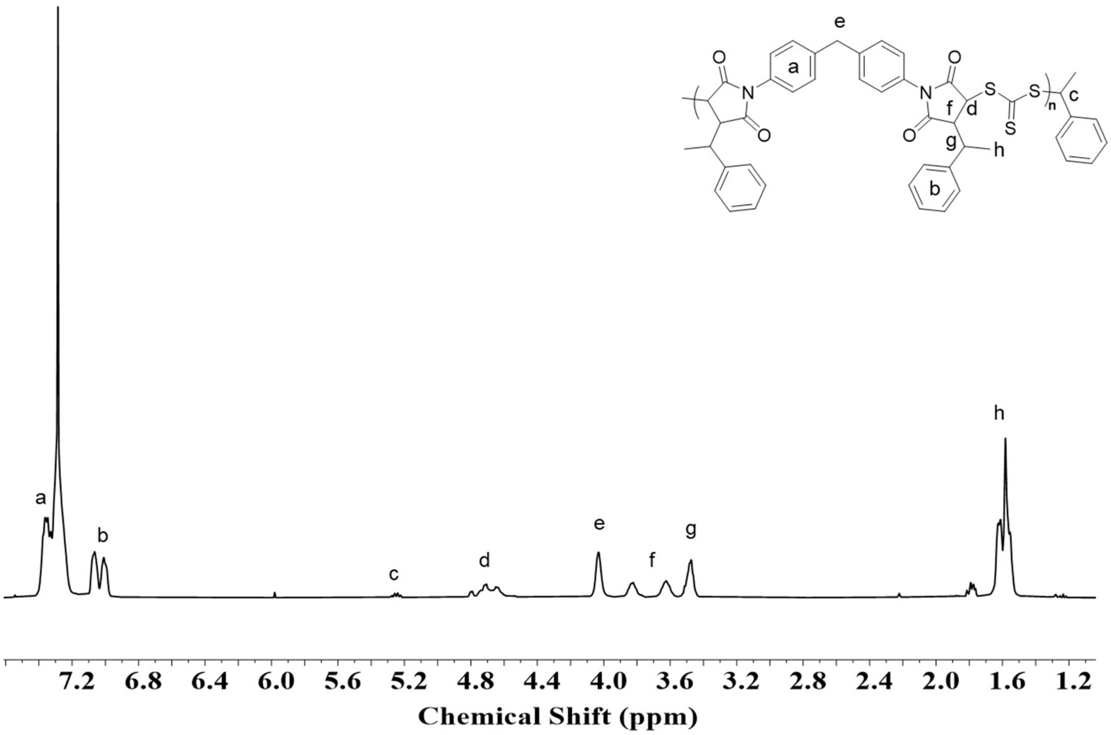

Figure S25. ^{1}H NMR spectra of the backbone (M_w = 12,100, $Đ$ = 2.46) in $CDCl_3$.

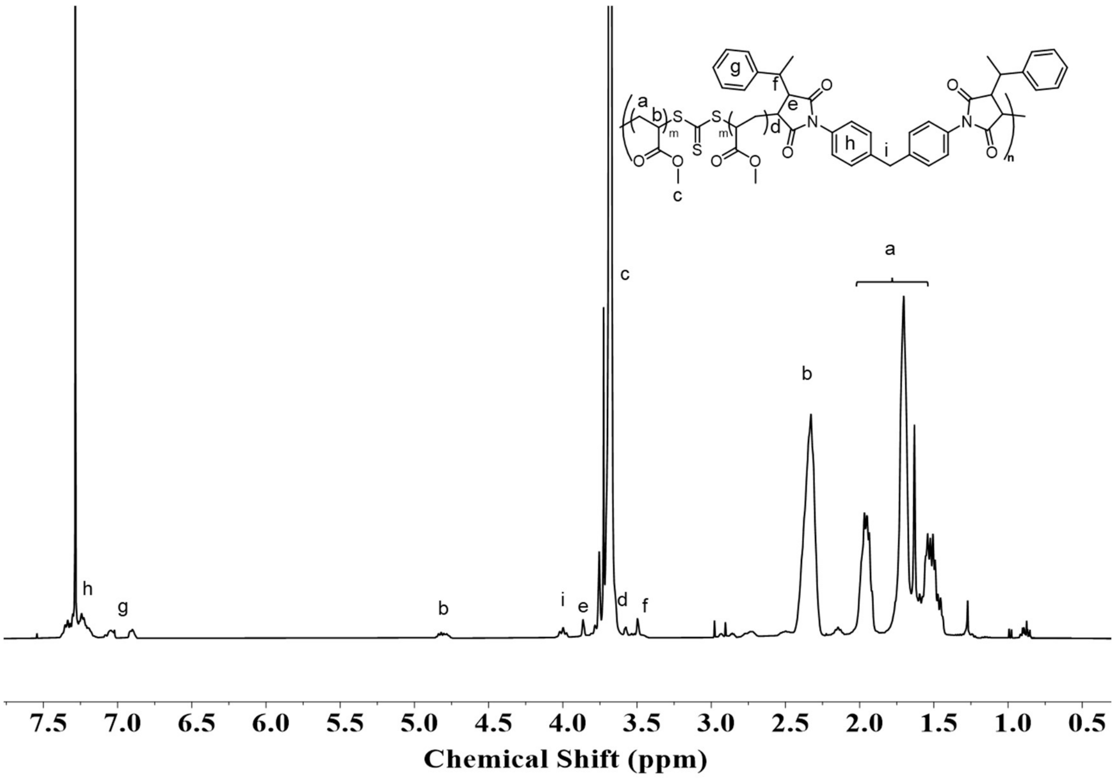

Figure S26. ^{1}H NMR spectra of PMA after chain expansion in **Figure 7A** in $CDCl_3$.

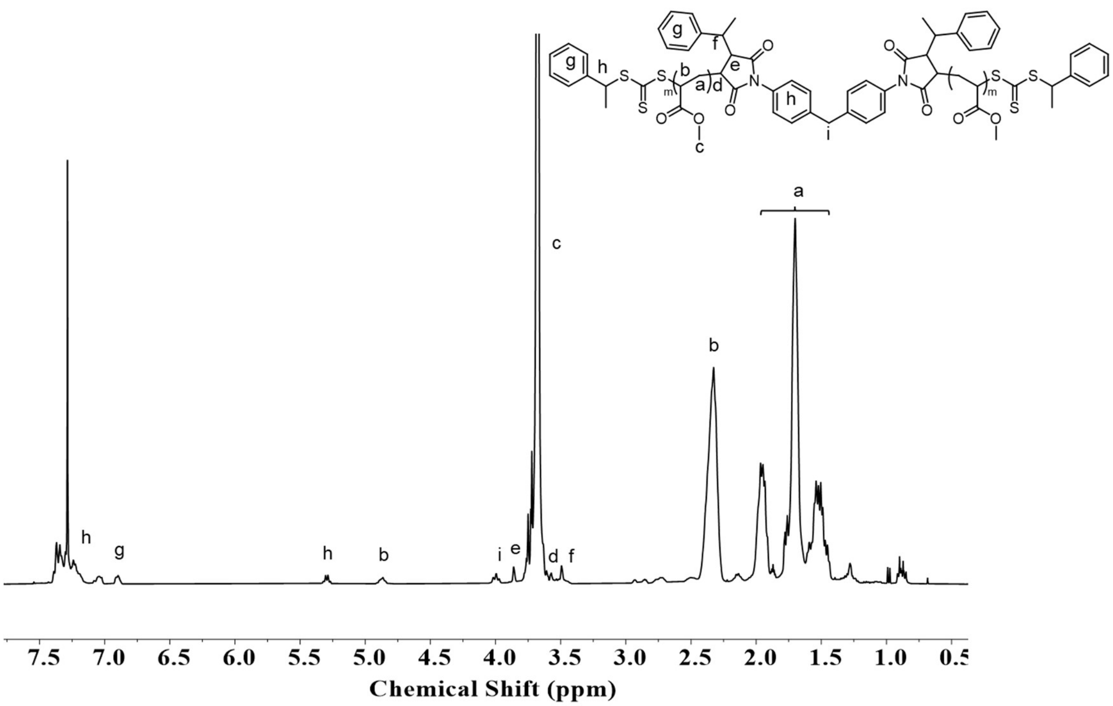

Figure S27. ^{1}H NMR spectra of PMA after deconstruction in **Figure 7A** in $CDCl_3$.

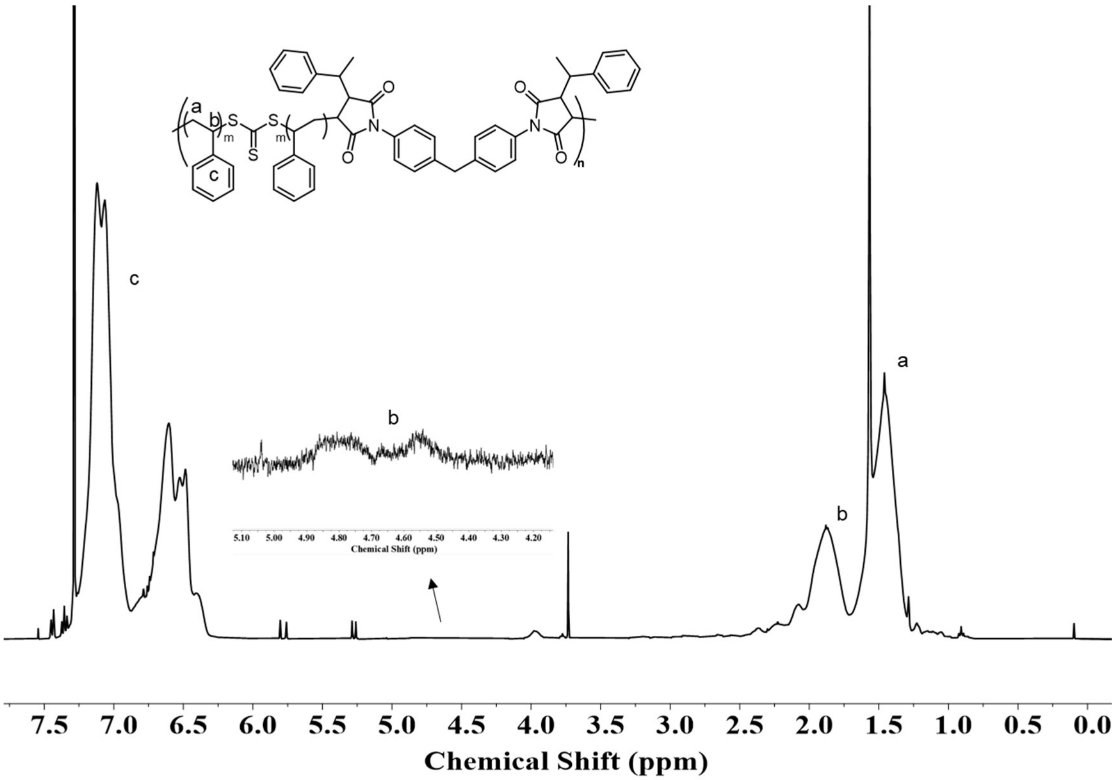

Figure S28. ^{1}H NMR spectra of PS after chain expansion in **Figure 7B** in $CDCl_3$.

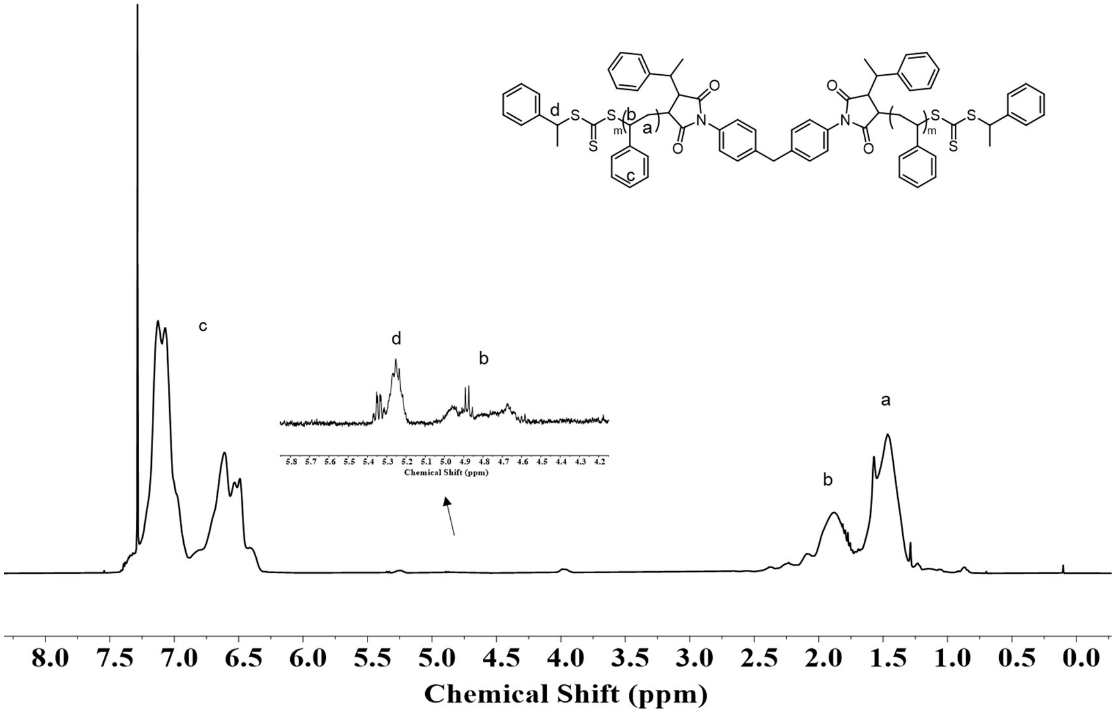

Figure S29. ^{1}H NMR spectra of PS after deconstruction in **Figure 7B** in $CDCl_3$.

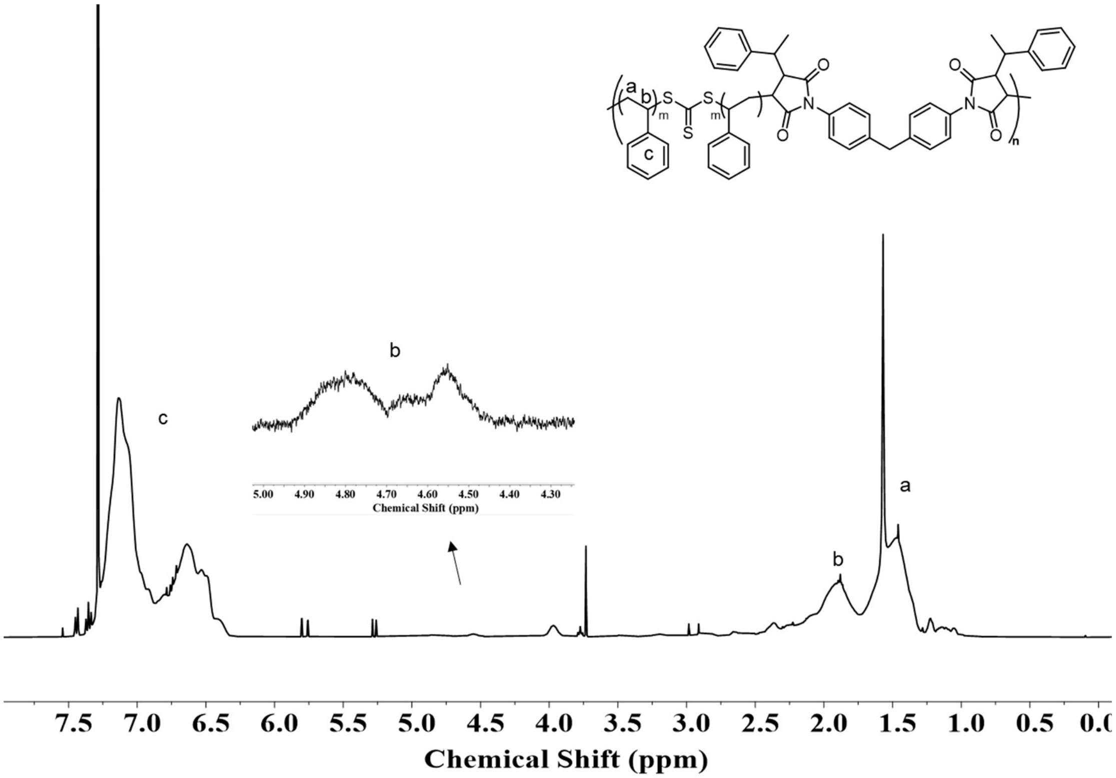

Figure S30. ^{1}H NMR spectra of PS after chain expansion in **Figure 8** in $CDCl_3$.

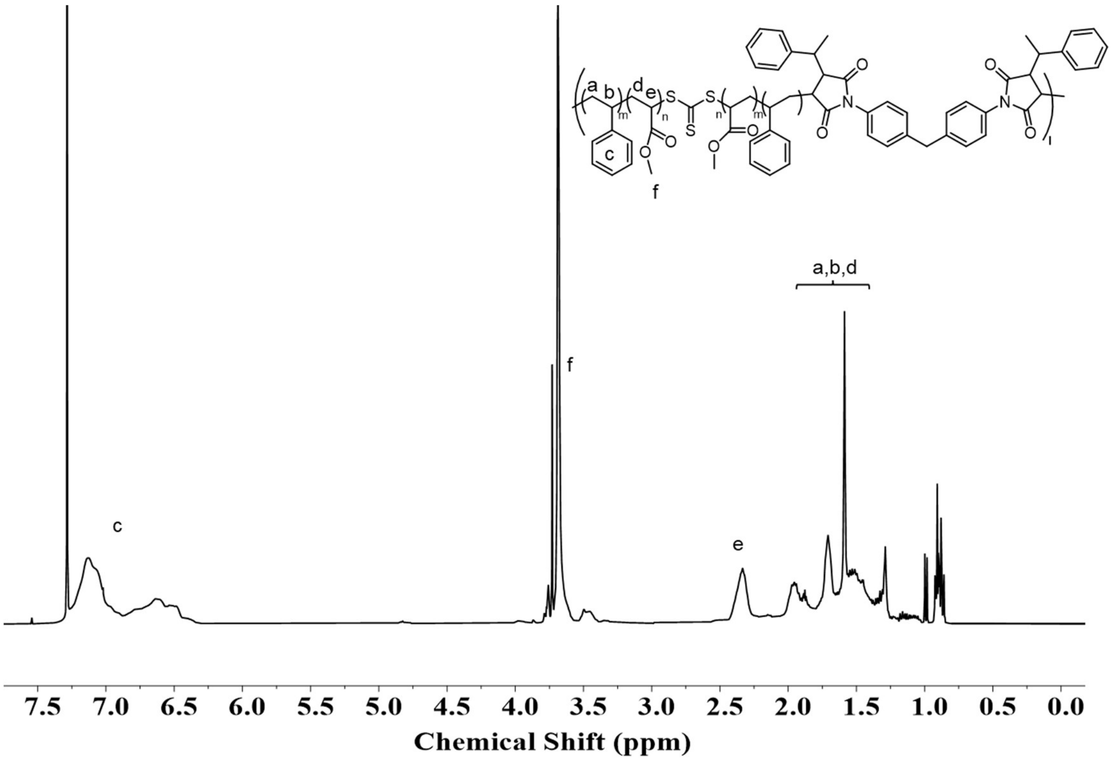

Figure S31. ^{1}H NMR spectra of the multiblock copolymers after chain expansion with MA in **Figure 8** in $CDCl_3$.

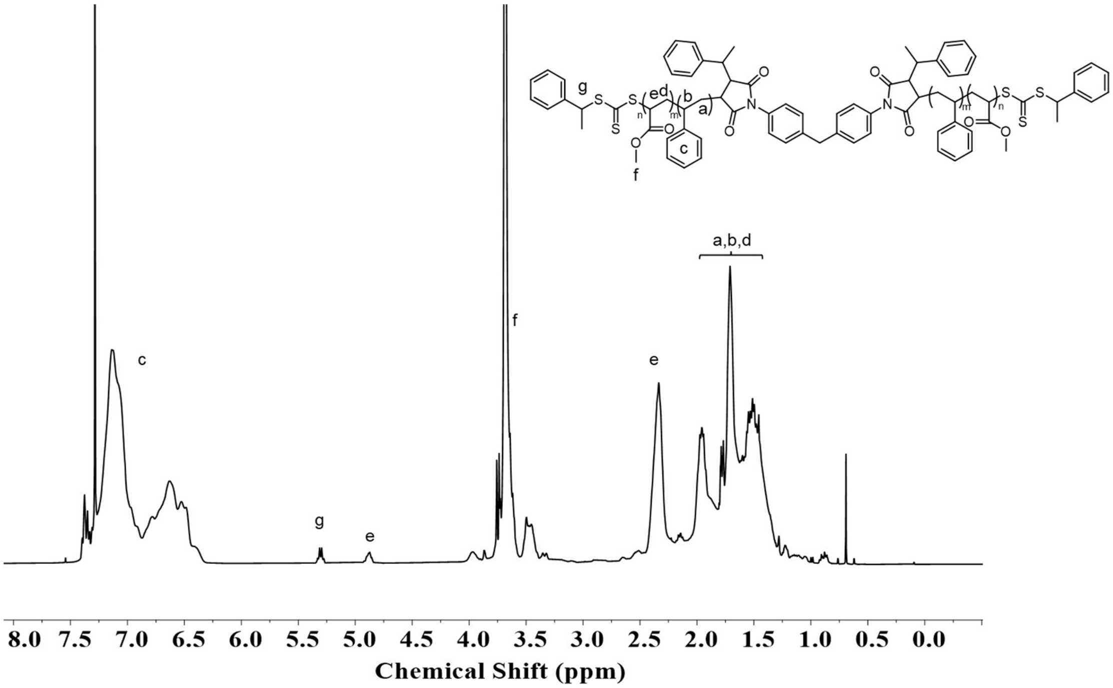

Figure S32. ^{1}H NMR spectra of PMA-*b*-PS-*b*-PMA after deconstruction in **Figure 8** in $CDCl_3$.

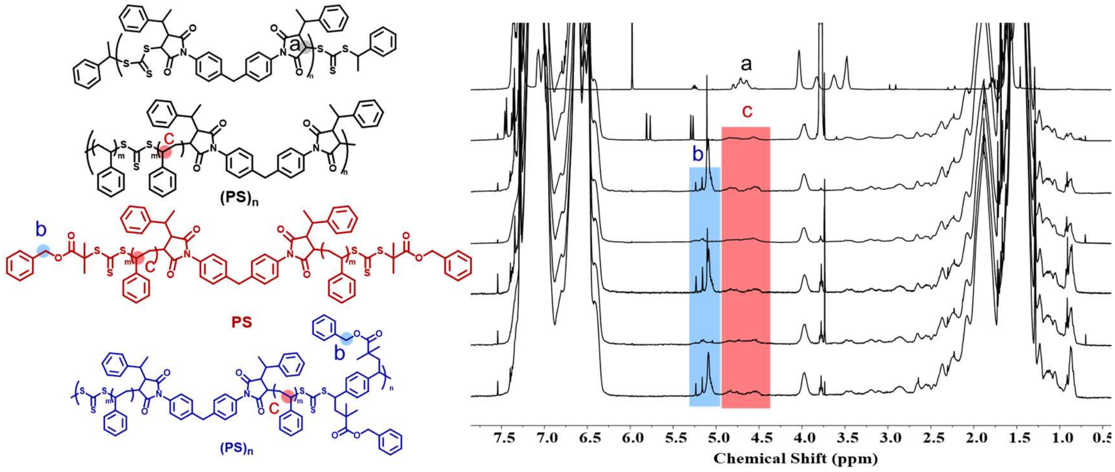

Figure S33. ^{1}H NMR spectra for second recycling of PS in **Figure 10** in $CDCl_3$.

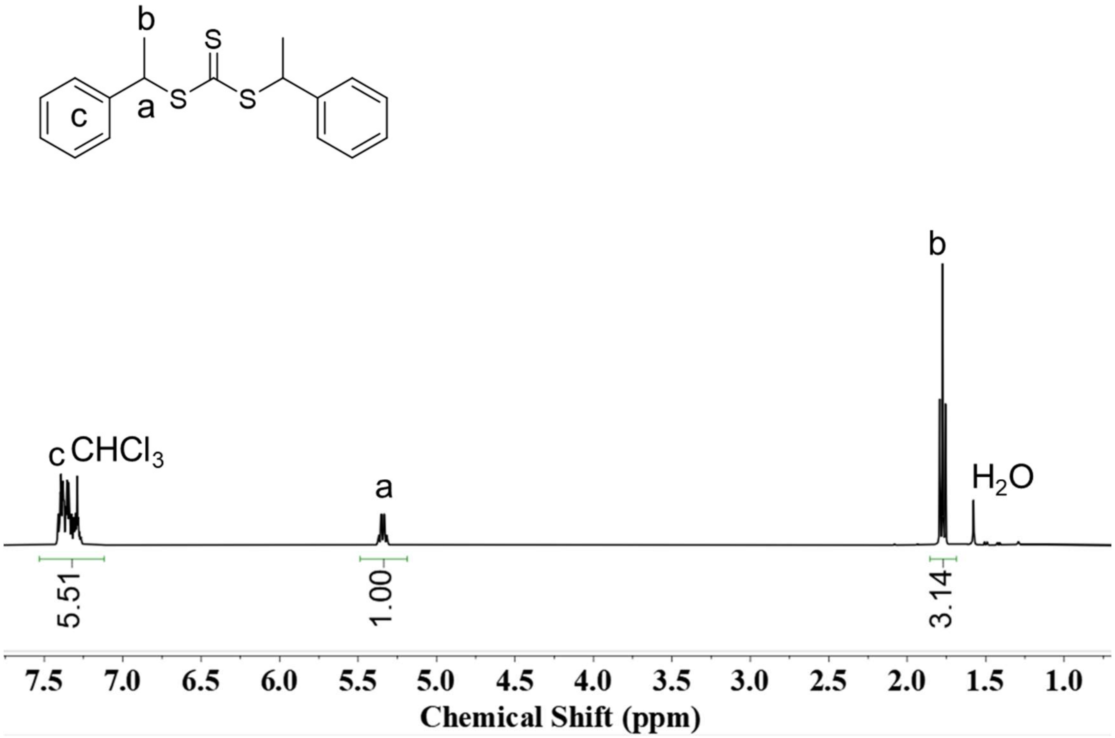

Figure S34. ^{1}H NMR spectrum of **CTA$_2$** in $CDCl_3$.

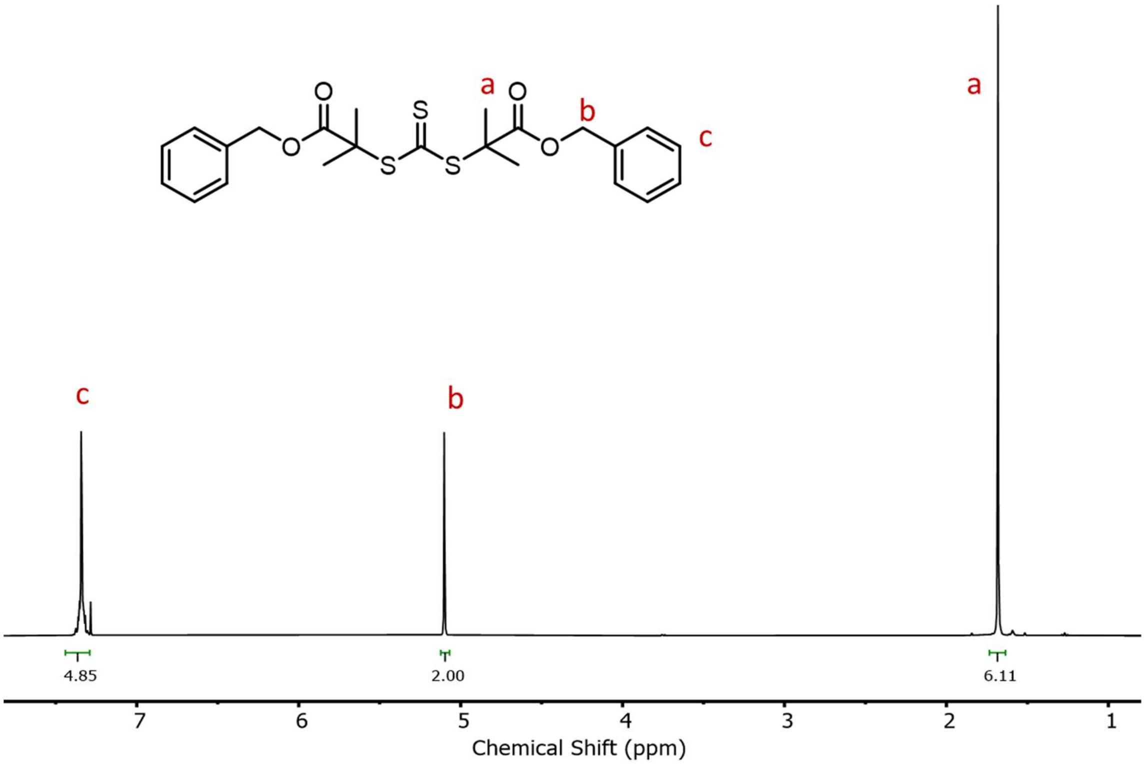

Figure S35. ^{1}H NMR spectrum of **CTA$_{2E}$** in $CDCl_3$.

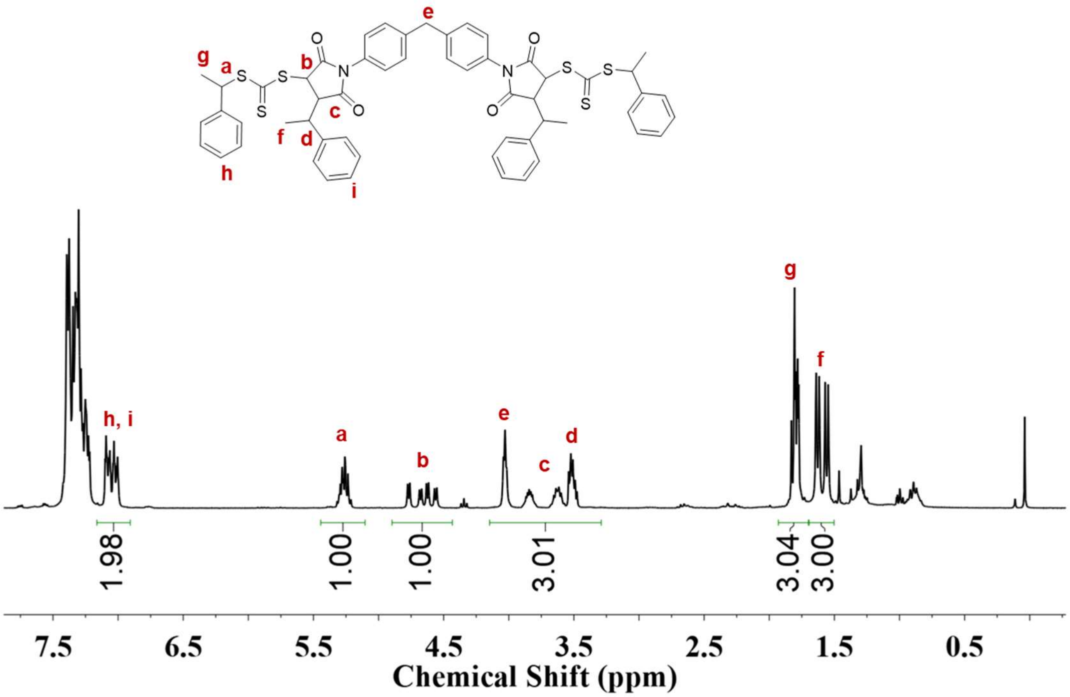

Figure S36. ^{1}H NMR spectrum in $CDCl_3$ of CTA_2-M_2-CTA_2 adduct purified after deconstruction of the backbone.

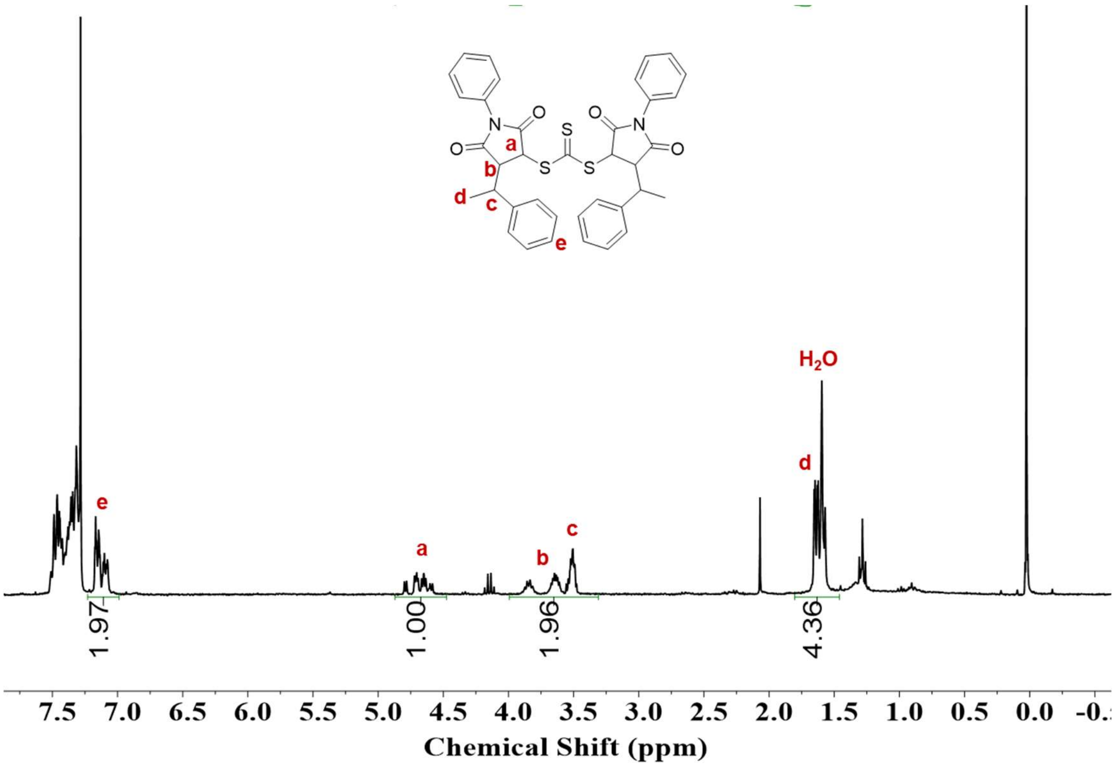

Figure S37. ^{1}H NMR spectrum in $CDCl_3$ of M-CTA_2-M adduct purified after RAFT-SUMI model reaction.

References:

1 P. J. Flory, *J. Am. Chem. Soc.* **1936**, *58*, 1877–1885.
2 J. Tanaka, N. E. Archer, M. J. Grant, W. You, *J. Am. Chem. Soc.* **2021**, *143*, 15918–15923.
3 T. Endo, N. Aoyagi, B. Ochiai, H. Mori, *Synlett* **2006**, 636–638.
